# Elements of the complete blood count associated with cardiovascular disease incidence: Findings from the EPIC-NL cohort study

**DOI:** 10.1038/s41598-018-21661-x

**Published:** 2018-02-19

**Authors:** Camille Lassale, Alyscia Curtis, Itziar Abete, Yvonne. T. van der Schouw, W. M. Monique Verschuren, Yunxia Lu, H. B(as). Bueno-de-Mesquita

**Affiliations:** 1 Department of Epidemiology and Public Health, University College London, 1-19 Torrington Place, London, WC1E 7HB United Kingdom; 2 Department of Epidemiology and Biostatistics, School of Public Health, Imperial College London, Norfolk Place, London, W2 1PG United Kingdom; 3 Nutrition Research Center, University of Navarra, 31010 Pamplona, Spain; 4 Julius Center for Health Sciences and Primary Care, University Medical Center Utrecht, Utrecht, The Netherlands; 5 Center for Nutrition, Prevention and Health Services, National Institute for Public Health and the Environment (RIVM), 3720 BA Bilthoven, The Netherlands; 6 Program in Public Health, College of Health Sciences, University of California Irvine, Irvine, CA United States of America; 7 Department of Molecular Medicine and Surgery, Karolinska Institutet, Stockholm, Sweden; 8 Department of Social & Preventive Medicine, Faculty of Medicine, University of Malaya, Pantai Valley, 50603 Kuala Lumpur Malaysia

## Abstract

All blood cells (white blood cells [WBC], red blood cells [RBC] and platelets) can play a role in atherosclerosis. Complete blood count (CBC) is widely available in clinical practice but utility as potential risk factors for cardiovascular disease (CVD) is uncertain. Our aim was to assess the associations of pre-diagnostic CBC with incidence of CVD in 14,362 adults free of CVD and aged 47.8 (±11.7) years at baseline, followed-up for 11.4 years (992 incident cases). Cox proportional hazards regressions were used to estimate HRs and 95%CI. Comparing the top (T3) to bottom (T1) tertile, increased total WBC, lymphocyte, monocyte and neutrophil counts were associated with higher CVD risk: 1.31 (1.10; 1.55), 1.20 (1.02; 1.41), 1.21 (1.03; 1.41) and 1.24 (1.05; 1.47), as well as mean corpuscular volume (MCV: 1.23 [1.04; 1.46]) and red cell distribution width (RDW: 1.22 [1.03; 1.44]). Platelets displayed an association for count values above the clinically normal range: 1.49 (1.00; 2.22). To conclude, total and differential WBC count, MCV, RDW and platelet count likely play a role in the aetiology of CVD but only WBC provide a modest improvement for the prediction of 10-year CVD risk over traditional CVD risk factors in a general population.

## Introduction

It is acknowledged that atherosclerosis, the underlying cause of ischemia, has inflammatory origin^1^. While biomarkers of inflammation are numerous, the count of white blood cells (WBC) has gained attention as an inexpensive test and various studies have consistently shown associations with increased risk of incident cardiovascular outcomes^2–4^. Less is known, however, about the role of WBC subtypes and of other components of the blood cell count such as red blood cell (RBC) and platelet characteristics. Nearly all the cellular elements in the blood are involved in the pathogenesis of atherosclerosis^1^. Regarding WBC subtypes, monocytes^5^, lymphocytes^6^ and neutrophils^7^ have been proposed as potentially better predictors of cardiovascular disease (CVD) risk than total WBC alone. As for RBC, associations of count^8^ or haematocrit^9,10^, mean corpuscular volume (MCV)^11^ and RBC distribution width (RDW)^12–15^ with cardiovascular risk have been reported in a variety of populations. RDW, a measure of the variability in size of circulating RBC commonly used for the diagnosis of anaemia, has recently drawn increased attention as a potential biomarker of CVD risk^12–15^. Platelets also play a key role in atherothrombosis^16^; hence platelet count, mean platelet volume (MPV) and distribution width (PDW) have been addressed as potential contributors of CVD risk but results are conflicting^17–19^. Most studies focused on patient populations with pre-existing CVD or looked at mortality rather than incidence of CVD. Therefore, more well-characterised and powered studies are needed to help clarify the potential role of blood count components as an inexpensive and routinely assessed set of biomarkers of CVD risk in previously healthy populations.

Our main objective was to explore the associations between complete blood cell counts and characteristics and risk of incident CVD and subtypes coronary heart disease and stroke over a long-term follow-up in a prospective cohort study, the European Prospective Investigation into Cancer and Nutrition-Netherlands (EPIC-NL) study. A secondary objective was to assess the incremental value for 10-year CVD risk prediction of complete blood count elements beyond well-established CVD risk factors.

## Methods

### Study population

The study population was derived from the MORGEN-EPIC and the Prospect-EPIC studies, conducted from 1993 up to 1997, which represent the Dutch part of the EPIC study. The cohort profile has been published elsewhere^20^. In brief, the MORGEN-EPIC cohort consists of men and women aged 20–65 years residing in Maastricht, Amsterdam, and Doetinchem. The Prospect-EPIC cohort consists of women 49–70 years, recruited from the national breast cancer screening program in Utrecht. Participants of both studies received two questionnaires (general and dietary) and were invited to attend a medical examination. The MORGEN-EPIC and Prospect-EPIC studies were approved by the medical ethical committees of the Netherlands Organisation for applied scientific research (TNO) and of the Academic Hospital Utrecht, respectively. Participants provided written informed consent. All methods were performed in accordance with the Declaration of Helsinki and guidelines and regulations of the TNO and Academic Hospital Utrecht ethics committees.

The initial sample was constituted of participants from EPIC-NL who had data on haematological parameters (n = 16,187). History of CVD (prevalent cases) was identified through linkage with the National Medical Registry from hospital discharge diagnosis database, and by self-report using the baseline questionnaire. We excluded participants with prevalent CVD at baseline (n = 227) and missing data on outcome (missing fatal or non-fatal CVD, n = 1249), leaving a sample of 14,772. We further excluded participants with missing data on smoking status (n = 48), physical activity (n = 32), educational level (n = 68), alcohol drinking (n = 62), body mass index (n = 2), blood pressure (n = 20), cholesterol (n = 226), and diabetes (n = 35). The final sample was formed of 14,362 individuals, including 5458 women from the Prospect cohort and 4066 men and 4833 women of the MORGEN cohort.

### Complete blood count measurement

During the medical examination upon entry into the cohort after June 1995, a blood sample was drawn of all subjects in an EDTA tube for measurement of the complete blood cell counts: WBC (total and subtypes), RBC and platelet counts. The storage process of blood samples was described previously^21^ and in Supplemental information. They were analysed on a blood cell counter (Coulter counter MAXM, Coulter Electronics). Short-term reliability was satisfactory and participation in the Interlaboratory Quality Assurance Program provided by Coulter Electronics showed that most measurements were valid^21^.The following parameters were considered as exposures: 1) RBC; count (10^12^cells/L), haematocrit (L/L), mean corpuscular volume (MCV, fL) and RDW (%) calculated as (standard deviation [SD] of MCV divided by MCV) x 100; 2) WBC; total and differential WBC count (10^9^ cells/L), including lymphocytes, monocytes and neutrophils; 3) Platelets; count (10^9^ cells/L), plateletcrit (L/L), mean platelet volume (MPV, fL) and platelet distribution width (PDW, %), calculated as (SD of MPV divided by MPV) x 100.

### Covariates

Data on education, smoking habits, alcohol consumption and physical activity were self-reported in the lifestyle questionnaire at baseline. Questions on occupational, recreational, and household physical activity during the past year were asked and the Cambridge Index of physical activity was derived by combining occupational with recreational activity, summarized into 4 groups: active, moderately active, moderately inactive, and inactive. This index has been validated and widely used in analyses in the EPIC study^22^. Smoking was categorized as never; former (quit smoking >20 years ago; quit 10–20 y ago; quit ≤10 y ago); current smoker (1–15 cigarettes/d; >16 cigarettes/d); pipe/cigar smoker. Level of education was categorized as low (primary education up to advanced elementary education), medium (intermediate vocational education and higher general secondary education) or high (higher vocational education and university). Alcohol intake was assessed by a simple frequency question “do you currently drink alcohol” and the answers were categorized into 3 groups (never, occasional [<1 drink/week] and frequent [≥1 drink/week drinker]). Anthropometric and blood pressure measurements were performed during the physical examination. Body mass index (BMI) was calculated as weight (kg) divided by the square of height (m^2^), and waist-to-hip ratio was the quotient between waist and hip circumference.

Blood pressure was the mean of two measures taken in a supine position on the right arm using a Boso Oscillomat (Bosch & Son) (Prospect) or on the left arm using a random zero Sphygmomano-meter (MORGEN). HDL and total cholesterol were determined with enzymatic assays. Diabetes was defined as a referral diagnosis or self-reported of type 2 diabetes, use of glucose-lowering agents, or a plasma glucose concentration of ≥7.0 mmol/L at baseline with initiation of glucose-lowering treatment within 1 year after inclusion. Anaemia was defined as haemoglobin <8.1 mmol/L (men) or <7.4 mmol/L (women)^23^.

### Outcome ascertainment

Vital status was identified using the municipal population register with a loss-to-follow-up of 2.6% and cause of death was obtained from Statistics Netherlands. Accuracy of cause of death claim in the register was assessed in a study where causes of death were coded again two years after initial coding, and agreement was 82% for coronary heart disease, and 79% for stroke^24^. Morbidity data were provided by the national hospital discharge register. Causes of death were coded according to the Tenth Revision of the International Statistical Classification of Diseases (ICD-10)^25^. Morbidity data were coded according to ICD-9. The outcome was first non-fatal or fatal CVD. Non-fatal CVD events were defined as ICD-9 codes 410–414, 427.5, 798.1,798.2, 798.9 (CHD), 428, 430–438 (stroke), 415.1, 443.9, 440–442, 444, and fatal CVD events by ICD-10 codes I20-I25, I46, R96 (CHD), G45, I60-I67, I69 (stroke), I26, I70-I74, I50. The definition of thee codes is given in Supplemental Table 1.

**Table 1:** Baseline characteristics by event type, the European Prospective Investigation into Cancer and Nutrition – Netherlands (EPIC-NL).

	All participants	CVD cases	Stroke cases	CHD cases
n	14362	992	196	589
Men (%)	28.3	35.0*	27.6	38.9*
Follow-up time (years), median (IQR)	11.4 (10.7;12.0)	6.8 (4.0;9.1)*	6.3 (3.7;8.7)*	6.4 (3.5; 8.9)*
Age (years), median (IQR)	50.2 (39.7; 56.4)	55.2 (50.0; 61.0)	56.8 (51.1; 64.7)	54.4 (49.7; 59.5)
Age (years), mean (SD)	47.8 (11.7)	54.7 (8.8)*	56.1 (9.8)*	54.3 (8.2)*
BMI (kg/m2), mean (SD)	25.5 (4)	26.9 (4.2)*	26.4 (4.4)*	27.2 (4.1)*
Waist to hip ratio, mean (SD)	0.83 (0.09)	0.86 (0.1)*	0.86 (0.09)*	0.87 (0.1)*
Educational level, (%)		*	*	*
Low	48.2	58.6	57.7	59.9
Medium	28.2	26.2	28.6	24.6
High	23.5	15.2	13.8	15.4
Physical activity, (%)		*	*	*
Inactive	7.2	12.8	10.7	14.3
Moderately inactive	24.6	28.2	24.5	28.5
Moderately active	26.5	23.1	32.1	21.4
Active	41.7	35.9	32.7	35.8
Smoking intensitiy, (%)		*	*	*
Never	37.6	27.6	31.1	26.5
Former, quit ≤10 y	9.5	10.1	10.7	9.7
Former, quit 11–20 y	10.1	10.2	9.2	10.5
Former, quit >20 y	11.1	10	8.2	11.7
Current, 1–15 cig/d	17.1	22.5	20.4	22.1
Current, ≥16cig/d	10.8	16.3	17.3	16.3
Current pipe/cigar or missing	3.9	3.3	3.1	3.2
Alcohol, (%)			*	
Non-drinker	8.3	9.1	7.1	10.4
Occasional drinker	26.7	28.8	35.7	27.7
Frequent drinker	65	62.1	57.1	62
**Complete blood count**				
*Red blood cell characteristics, mean (SD)*				
RBC(10^12^ cells/L)^a^	4.6 (0.5)	4.7 (0.5)*	4.7 (0.5)	4.7 (0.5)*
Hematocrit (L/L)	0.42 (0.04)	0.43 (0.04)*	0.43 (0.04)*	0.43 (0.04)*
MCV (fL)^b^	90.9 (4.6)	91.5 (4.8)*	92.0 (4.0)*	91.3 (5.1)
RDW (%)^c^	12.3 (1.8)	12.4 (0.9)*	12.4 (0.8)	12.4 (1)*
*White blood cell characteristics, mean (SD)*				
WBC (10^9^ cells/L)^d^	6.7 (1.9)	7.2 (2.1)*	7.2 (2.1)*	7.1 (2.1)*
Lymphocytes (10^9^ cells /L)	1.9 (0.6)	2.1 (0.8)*	2.0 (0.7)	2.1 (0.8)*
Monocytes (10^9^ cells /L)	0.47 (0.2)	0.51 (0.25)*	0.52 (0.37)*	0.51 (0.21)*
Neutrophils (10^9^ cells /L)	4 (1.6)	4.3 (1.6)*	4.5 (1.7)*	4.3 (1.6)*
*Platelet characteristics, mean (SD)*				
Platelet (10^9^ cells /L)	253.7 (59.9)	256.5 (64.7)	255.9 (63.7)	256.9 (64.3)
Platetetcrit (L/L)	0.23 (0.19)	0.23 (0.05)	0.23 (0.06)	0.23 (0.05)
MPV (fL)^e^	9.1 (1.1)	9.1 (1.1)	9.1 (1)	9.1 (1.1)
PDW (%)^f^	15.8 (1.7)	15.8 (0.5)	15.8 (0.5)	15.8 (0.5)

^a^Red blood cells count; ^b^Mean corpuscular volume; ^c^Red cell distribution width; ^d^White blood cells count; ^e^Mean platelet volume; ^f^Platelet distribution width; *Significant difference between cases and non-cases (p < 0.05). The p-value is obtained by t-test for continuous variables and by chi-square test for categorical variables.

### Statistical analyses

Baseline characteristics were compared by t-test and chi-square test (for continuous and categorical variables, respectively), between participants with and without the outcome of interest. The proportional hazards assumption was tested for each haematological parameter based on the Schoenfeld residuals, showing no violation of the assumption. To estimate hazard ratios (HRs) and 95% confidence intervals of CVD, we used Cox proportional hazards regression models, with age as the underlying time scale. For the analysis on CVD incidence, exit age was at the time of first fatal or non-fatal CVD event, loss to follow-up or 31 December 2012, whichever came first. A competing risk model was fitted to estimate cause-specific sub hazard ratios^26^ for CHD and stroke, with the other outcome taken as a competing risk in the analysis. All models were stratified by sex and cohort, to account for the difference in baseline hazard between men and women and in the different centres, particularly due to the difference in age between the two cohorts.

In Model 1, we adjusted for age, smoking status and intensity, BMI, waist-to-hip ratio, education, physical activity, and alcohol consumption as these are modifiable risk factors associated with CVD risk. In Model 2, we further adjusted for systolic blood pressure, HDL cholesterol and diabetes, which are known CVD risk markers. For RBC count, models were further adjusted for haemoglobin as a marker of anaemia. For RDW, we further adjusted for haemoglobin, white blood cell count and platelet count^14,15^. For PDW, we further adjusted for platelet count. To assess the relative compared to the absolute increase in each WBC subtype (lymphocytes, monocytes, neutrophils) counts, we further adjusted for total WBC in a separate model.

Analyses were conducted per sex-specific tertiles, as well as pre-defined categories (clinically relevant) of each parameter. Linear trend across tertiles was tested by modelling a 3-level ordinal variable that took the value of the median in each tertile, and computing a p-value for the coefficient of that variable. Interactions with sex and smoking, as well as anaemia status for RBC analyses, were explored by modelling the cross-product terms in the fully adjusted models. In particular, stratification rather than adjustment can help disentangle the association between blood cells and CVD risk independently of smoking, because smoking is so strongly related to both exposure and outcome that residual confounding may still occur when adjusting for smoking status and intensity. Sensitivity analyses were conducted by (1) excluding prevalent cancer cases at baseline due to the potential modifications of the blood count by cancer^27^ and the increased cardiovascular risk of cancer patients^28^; (2) excluding participants with less than two years of follow-up to avoid reverse causality, as a change in blood count may be the consequence rather than the cause of events that would occur shortly after the measurement and (3) repeating the analyses with cohort- and sex-specific tertiles.

A secondary objective was to assess the added predictive performance of complete blood count elements to an established CVD risk prediction model. We chose the SCORE equation^29^, recommended by the latest European Society of Cardiology 2016 Guidelines on CVD prevention in clinical practice^30^. Therefore, our base model included sex (stratified), age, smoking status (binary), SBP and the ratio of total- to HDL-cholesterol, with time to follow-up as underlying time variable in the Cox model. We estimated the Harrell’s C-statistic as a measure of discrimination in the base model and the change in the C-statistic after inclusion of each of the significant complete blood count elements. We also assessed reclassification by the means of the continuous net reclassification improvement (NRI) and integrated discrimination improvement (IDI) using 3 categories of 10-year risk (0–5%, 5–10%, ≥10%).

All tests were two-sided at the 0.05 level and the main analyses were conducted using SAS 9.3 (Cary, NC). Discrimination and reclassification analyses were conducted in STATA 14, using the programs developed by the Cardiovascular Epidemiology Unit at the University of Cambridge, predaddc and predstat (http://www.phpc.cam.ac.uk/ceu/erfc/programs/).

### Data availability

For information on how to submit an application for gaining access to EPIC data and/or biospecimens, please follow the instructions at http://epic.iarc.fr/access/index.php.

## Results

Among the 14,362 participants (28% men) with a mean (SD) age of 47.8 (11.7) years, there were 992 incident CVD cases of which 196 were stroke and 589 were CHD. In total, 156,589 person-years were followed-up for a median of 11.4 years. Lifestyle characteristics are presented in Table 1. Compared to participants without the outcome of interest, incident CVD cases were older, more often men, had a higher BMI and waist-to-hip ratio, were less physically active and more often smokers. All RBC and WBC characteristics differed between cases and non-cases, whereas differences in platelet characteristics were non-significant.

### RBC

**Figure 1:**
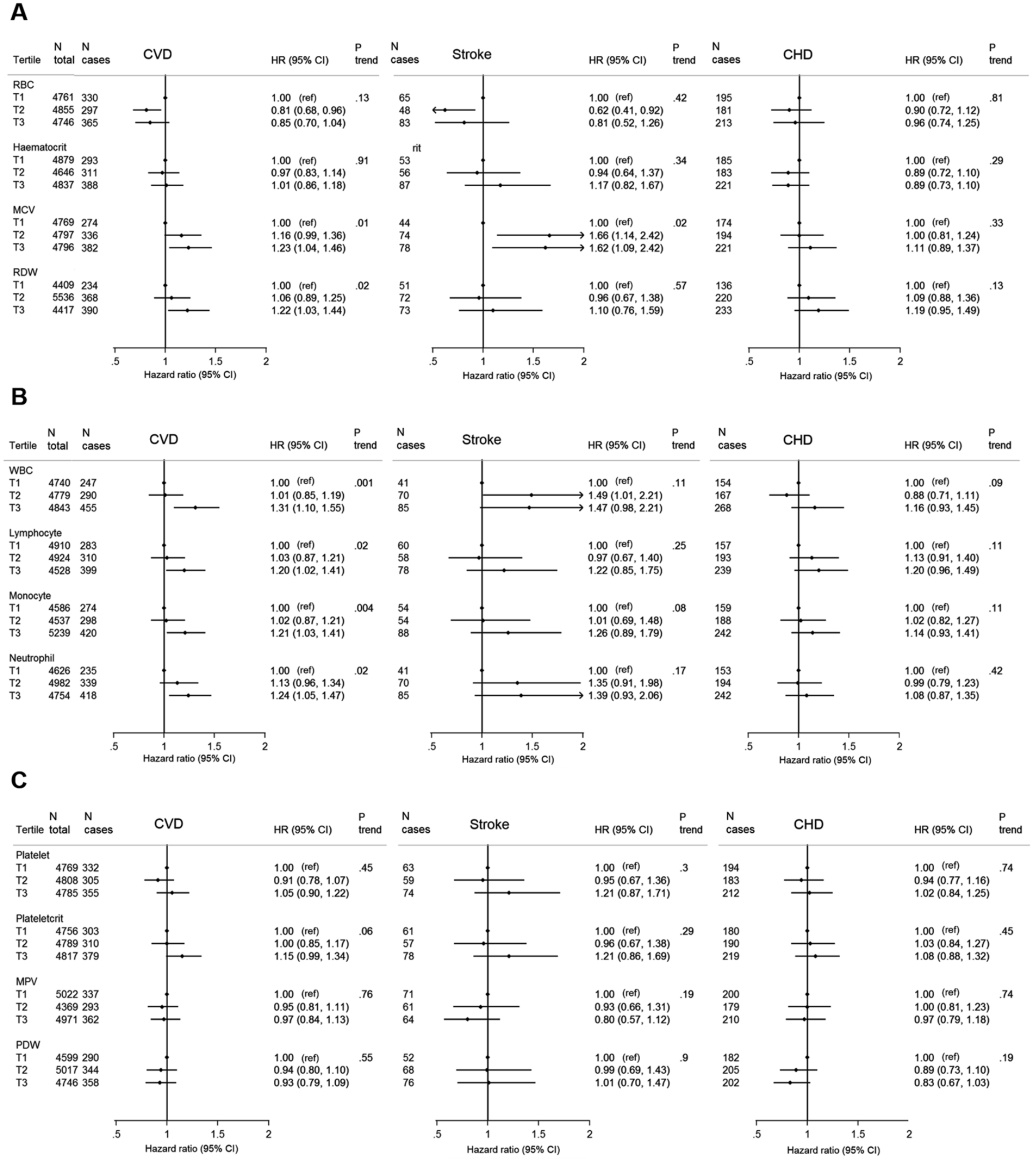
Multivariate (Model 2) hazard ratios HRs and 95% confidence interval for CVD associated with tertiles of red blood cell parameters (panel A), white blood cell parameters (panel B) and platelet parameters (panel C), n = 14,362, EPIC-NL. HRs adjusted for age, smoking status and intensity (7 categories), body mass index (BMI, continuous), Waist-to-hip ratio (WHR, continuous), physical activity level (Cambridge index, 4 categories), educational level (low, medium, high), alcohol intake (non drinker, occasional drinker, frequent drinker), systolic blood pressure, HDL cholesterol and diabetes. Stratified by sex and cohort centre. Age is the underlying time variable. Exit age is age at first outcome of interest or censoring. Cut-off points for tertiles: Men: RBC 4.9, 5.2; Haematocrit 0.45, 0.47; MCV 89.7, 92.7; RDW 12, 12.4; WBC 5.7, 7.0; Lymphocyte 1.7, 2.1; Monocyte 0.5, 0.6; Neutrophils 3.1, 4.1; Platelets 212, 253; Plateletcrit 0.19, 0.23; MPV 8.6, 9.3; PDW 15.6, 15.9; Women: RBC 4.3, 4.6; Haematocrit 0.39,0.42; MCV 89.3, 92.5; RDW 11.9, 12.4; WBC 5.7, 7.1; Lymphocyte 1.7, 2.1; Monocyte 0.4, 0.5; Neutrophils 3.3, 4.4; Platelets 233, 279; Plateletcrit 0.21, 0.25; MPV 8.7, 9.4; PDW 15.5, 15.8.

**Figure 2:**
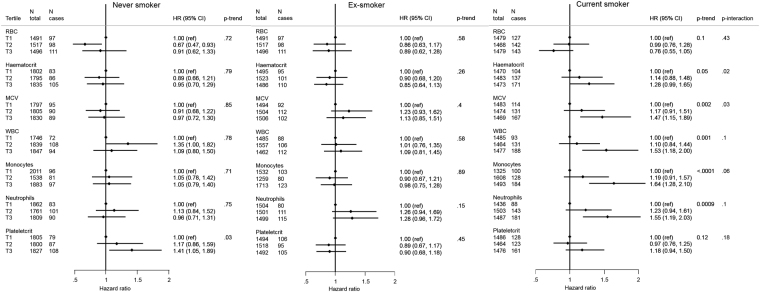
Multivariate (Model 2) hazard ratios and 95% confidence interval for CVD per tertile of selected elements of the complete blood count, by smoking status at baseline, n = 14,362, EPIC-NL. HRs adjusted for age, smoking intensity, body mass index (BMI, continuous), Waist-to-hip ratio (WHR, continuous), physical activity level (Cambridge index, 4 categories), educational level (low, medium, high), alcohol intake (non drinker, occasional drinker, frequent drinker), systolic blood pressure, HDL cholesterol and diabetes. Stratified by sex and cohort centre. Age is the underlying time variable. Exit age is age at first outcome of interest or censoring.

Neither RBC count nor haematocrit were linearly associated with risk of CVD, stroke or CHD in both models (Fig. 1, Supplemental Table 2). When participants with a baseline diagnosis of cancer were excluded, haematocrit was linearly associated with total CVD and stroke risk in Model 1 (Supplemental Table 3) but not when further adjusted for other CVD risk factors (Supplemental Table 4). MCV and RDW were both associated with CVD risk, particularly in Model 2 (Fig. 1A). Higher stroke risk was also observed at higher MCV values (Fig. 1A), whereas higher CHD risk was observed at very low values (Table 2). Associations with haematocrit and MCV with higher risk of CVD were modified by smoking status (p-interaction = 0.02 and 0.03 respectively) and were significant only in current smokers (Fig. 2). Whilst there was no significant interaction between RBC count and smoking, there was a negative association comparing the middle to the bottom RBC tertile only in never smokers. We identified 374 individuals with anaemia and there was no significant interaction between RBC count and anaemia (p = 0.68), nor meaningful differences when stratifying analyses by anaemia status.

**Table 2:** Multivariate (Model 2^a^) hazard ratios of CVD, stroke and CHD by clinically defined categories of elements of the complete blood count, n = 14,362, EPIC-NL.

	Category	N total	N cases	CVD			Stroke		N cases	CHD	
				HR (95% CI)	P-trend	N cases	HR (95% CI)	P-trend		HR (95% CI)	P-trend
**RBC**											
RBC (10^12^ cells/L)	Low (<4.5)M & (<3.8)F	482	42	1.32 (0.95; 1.85)	0.18	10	2.13 (1.05; 4.30)	0.04	22	0.99 (0.62; 1.58)	0.25
	Normal (4.5–6.5)M & (3.8–5.8)F	13872	949	1 (ref)		186	1 (ref)		566	1 (ref)	
	High (≥6.5)M & (≥5.8)F	8	1	2.29 (0.32; 16.46)		0	NA		1	5.37 (0.74; 38.74)	
Haematocrit (L/L)	Low (<0.40)M & (<0.37)F	1146	59	1.09 (0.83; 1.42)	0.16	8	0.70 (0.34; 1.43)	0.36	40	1.38 (0.99; 1.91)	0.14
	Normal (0.40–0.52)M & (0.37–0.47)F	13039	908	1 (ref)		182	1 (ref)		535	1 (ref)	
	High (≥0.52)M & (≥0.47)F	177	25	1.45 (0.97; 2.17)		6	1.54 (0.67; 3.50)		14	1.22 (0.68; 2.17)	
Mean cell volume (fL)	Low (<77)	133	11	1.64 (0.90; 2.99)	0.01	0	NA	0.39	9	2.27 (1.16; 4.42)	0.02
	Normal (77–95)	12033	781	1 (ref)		157	1 (ref)		467	1 (ref)	
	High (≥95)	2196	200	1.25 (1.06; 1.47)		39	1.18 (0.81; 1.72)		113	1.18 (0.94; 1.47)	
RDW (%)	Low (<11.5)	1125	49	0.83 (0.62; 1.11)	0.05	12	1.00 (0.55; 1.80)	0.51	27	0.74 (0.49; 1.11)	0.21
	Normal (11.5–15)	13037	926	1 (ref)		181	1 (ref)		552	1 (ref)	
	High (≥15)	200	17	1.73 (1.03; 2.92)		3	2.03 (0.61; 6.72)		10	1.44 (0.72; 2.89)	
**WBC**											
WBC (10^9^ cells)	Low (<4.5)	1139	49	0.80 (0.59; 1.07)	0.01	8	0.64 (0.31; 1.31)	0.03	28	0.82 (0.56; 1.20)	0.40
	Normal (4.5–10)	12397	851	1 (ref)		165	1 (ref)		513	1 (ref)	
	High (> = 10)	826	92	1.34 (1.07; 1.69)		23	1.75 (1.10; 2.79)		48	1.15 (0.84; 1.58)	
Lymphocyte (10^9^ cells)	Low (<1.3)	1390	81	0.96 (0.76; 1.22)	0.26	19	1.11 (0.68; 1.81)	0.91	45	0.94 (0.69; 1.29)	0.83
	Normal (1.3–3.5)	12659	871	1 (ref)		171	1 (ref)		522	1 (ref)	
	High (> = 3.5)	313	40	1.30 (0.94; 1.80)		6	0.97 (0.42; 2.21)		22	1.11 (0.71; 1.75)	
Monocyte (10^9^ cells)	Low <0.2	173	8	0.64 (0.32; 1.28)	0.0002	2	0.73 (0.18; 2.97)	0.32	5	0.73 (0.30; 1.77)	0.06
	Normal 0.2–0.8	13181	860	1 (ref)		172	1 (ref)		512	1 (ref)	
	High> = 0.8	1008	124	1.48 (1.22; 1.81)		22	1.40 (0.88; 2.23)		72	1.35 (1.04; 1.76)	
Neutrophil (10^9^ cells)	Low < 2.0	423	21	1.09 (0.71; 1.69)	0.82	2	0.53 (0.13; 2.15)	0.18	14	1.29 (0.75; 2.19)	0.58
	Normal 2.0–7.5	13460	931	1 (ref)		182	1 (ref)		555	1 (ref)	
	High >7.5	479	40	1.08 (0.78; 1.50)		12	1.64 (0.90; 3.01)		20	0.89 (0.56; 1.42)	
**Platelets**											
Platelet count (10^9^ cells/L)	Low <150	299	23	1.01 (0.66; 1.52)	0.15	8	1.91 (0.93; 3.93)	0.21	13	0.74 (0.39; 1.39)	0.07
	Normal 150–400	13818	944	1 (ref)		184	1 (ref)		559	1 (ref)	
	High > = 400	245	25	1.49 (1.00; 2.22)		4	1.16 (0.43; 3.15)		17	1.69 (1.03; 2.80)	
Plateletcrit (L/L)	Low <0.15	470	29	0.84 (0.58; 1.22)	0.31	9	1.33 (0.67; 2.61)	0.72	19	0.85 (0.52; 1.38)	0.16
	Normal 0.15–0.40	13822	955	1 (ref)		186	1 (ref)		564	1 (ref)	
	High > = 0.40	70	8	1.54 (0.77; 3.10)		1	0.98 (0.14; 7.02)		6	2.08 (0.93; 4.68)	
Mean platelet volume (fL)	Low <7.5	468	39	1.27 (0.92; 1.76)	0.29	5	0.81 (0.33; 1.97)	0.73	23	1.30 (0.85; 1.97)	0.38
	Normal 7.5–11.5	13543	923	1 (ref)		187	1 (ref)		547	1 (ref)	
	High > = 11.5	351	30	1.12 (0.78; 1.61)		4	0.72 (0.27; 1.94)		19	1.18 (0.74; 1.90)	

^a^HRs adjusted for age, smoking status and intensity (7 categories), body mass index (BMI, continuous), Waist-to-hip ratio (WHR, continuous), physical activity level (Cambridge index, 4 categories), educational level (low, medium, high), alcohol intake (non drinker, occasional drinker, frequent drinker), systolic blood pressure, HDL cholesterol and diabetes. Stratified by sex and cohort center. Age is the underlying time variable. Exit age is age at first outcome of interest or censoring. CVD, cardiovascular disease; CHD, coronary heart disease.

**Table 3:** Multivariate (Model 2^a^) hazard ratios per tertile of red blood cell and platelet distribution width, separately for men and women, n = 14,362, EPIC-NL.

	Tertile	Men				Women			P trend	p-interaction
		N total	N cases	HR (95%CI)	P trend	N total	N cases	HR (95%CI)		
**CVD**										
RDW (%)^d^	T1	1328	73	1 (ref)	0.07	3081	161	1 (ref)	0.26	0.0004
	T2	1485	123	1.22 (0.91; 1.63)		4051	245	0.97 (0.79; 1.19)		
	T3	1253	151	1.33 (0.99; 1.79)		3164	239	1.16 (0.93; 1.44)		
PDW (%)^f^	T1	1336	86	1 (ref)	0.14	3263	204	1 (ref)	0.09	0.007
	T2	1389	131	1.30 (0.99; 1.72)		3628	213	0.78 (0.64; 0.96)		
	T3	1341	130	1.28 (0.96; 1.71)		3405	228	0.80 (0.65; 0.99)		
**CHD**										
RDW (%)^d^	T1	1328	45	1 (ref)	0.10	3081	91	1 (ref)	0.32	0.005
	T2	1485	89	1.45 (0.99; 2.10)		4051	131	0.92 (0.70; 1.21)		
	T3	1253	95	1.33 (0.90; 1.96)		3164	138	1.11 (0.84; 1.48)		
PDW (%)^f^	T1	1336	51	1 (ref)	0.07	3263	131	1 (ref)	0.002	0.0003
	T2	1389	90	1.48 (1.04; 2.12)		3628	115	0.67 (0.52; 0.87)		
	T3	1341	88	1.44 (0.99; 2.09)		3405	114	0.63 (0.48; 0.82)		

^a^HRs adjusted for age, smoking status and intensity (7 categories), body mass index (BMI, continuous), Waist-to-hip ratio (WHR, continuous), physical activity level (Cambridge index, 4 categories), educational level (low, medium, high), alcohol intake (non drinker, occasional drinker, frequent drinker), systolic blood pressure, HDL cholesterol and diabetes. Stratified by sex and cohort centre. Age is the underlying time variable. Exit age is age at first outcome of interest or censoring. Abbreviations: CVD, cardiovascular disease; CHD, coronary heart disease.

**Table 4:** Change in discrimination (C-statistic) and reclassification for the evaluation of incremental value of selected elements of the complete blood count over the SCORE risk prediction model.

	C-statistic	95% CI		p-value difference	Categorical NRI	95% CI		p-value	IDI	95% CI		p-value
Model 0 “SCORE”^a^	0.7324	0.7168	0.748		Ref				Ref			
Model 0 + MCV	0.7330	0.7174	0.7486	0.25	0.51%	−0.82%	1.84%	0.45	0.0005	−0.0001	0.0010	0.08
Model 0 + RDW	0.7325	0.7168	0.7481	0.41	0.00%	−0.34%	0.34%	1.00	0.0000	0.0000	0.0001	0.15
Model 0 + WBC	0.7345	0.719	0.7501	0.14	0.50%	−1.70%	2.71%	0.65	0.0034	0.0018	0.0051	<0.001
Model 0 + Lymphocytes	0.7348	0.7193	0.7503	0.02	1.08%	−0.82%	2.99%	0.27	0.0017	0.0001	0.0033	0.03
Model 0 + Monocytes	0.7346	0.7191	0.7501	0.14	1.24%	−0.80%	3.28%	0.23	0.0024	0.0010	0.0038	0.001
Model 0 + Neutrophils	0.7329	0.7173	0.7486	0.63	−0.72%	−2.63%	1.19%	0.46	0.0020	0.0009	0.0032	0.001
Model 0 + Platelet count	0.7329	0.7173	0.7485	0.52	−0.43%	−2.05%	1.19%	0.60	0.0005	−0.0002	0.0011	0.20
Model 0 + PDW	0.7327	0.7171	0.7483	0.32	−0.17%	−1.21%	0.87%	0.76	0.0000	−0.0002	0.0002	0.99

^a^Model 0 includes age, smoking status (binary), total- to HDL-cholesterol ratio, systolic blood pressure and is stratified by sex and cohort. The primary time variable is follow-up time. Abbreviations: NRI, net reclassification improvement, categorical (three 10-year risk categories: 0–5%, 5–10%, >10%); IDI, integrated discrimination improvement.

### WBC

An increased CVD risk was observed at elevated counts of total WBC and subtypes (Fig. 1B) and the HRs for total WBC, monocyte and neutrophil counts were strongest amongst current smokers, but absent in never smokers (Fig. 2). The effect of total WBC was stronger for risk of stroke than of CHD. Lymphocyte count was associated with CVD risk (p-trend = 0.02) and not modified by smoking status. When assessing the relative increase of each subtype by adjusting for total WBC, all HRs became non-significant (Supplemental Table 5).

### Platelets

No association was found with platelets in the analyses per tertiles (Fig. 1C). However, though based on small numbers, increased risks were observed at levels above the clinically defined “normal range” for both platelet count (risks of CVD and CHD) and plateletcrit (risk of CHD) (Table 2). Smoking status modified the association with plateletcrit: an increased CVD risk at higher plateleletcrit values was only observed in never smokers (Fig. 2). Interestingly, sex was an effect modifier of the otherwise null association with PDW: greater PDW was associated with increased CVD and CHD risk in men, but with a decreased risk in women (Table 3).

The use of cohort-specific tertiles did not change substantially the associations (Supplemental Table 8) and observations were robust to other sensitivity analyses (Supplemental Tables 3, 4, 6 and 7).

### Predictive performance

The addition of each of the complete blood count elements significantly associated with CVD incidence to a base risk prediction model including the SCORE variables resulted in only little improvement in predictive performance, with only slight improvements observed for WBC (Table 4). The base model showed adequate discrimination with a C-statistic of 0.7324. An improvement in discrimination was observed for lymphocytes (change in C-statistic +0.0024, p = 0.02) but the very modest positive changes observed for the other elements were not statistically significant. In terms of reclassification into 10-year risk categories (0–5%, 5–10%, >10%), the categorical NRI was non-significant for all elements but the IDI was positive and significant for all WBC counts (total and differential).

## Discussion

We aimed to identify components of the complete blood count, which are reported in routine clinical practice, associated with long-term risk of CVD. This is the first population-based prospective study to assess the associations and predictive value of all elements of the complete blood count with incident CVD. We found that increased total WBC, lymphocyte, monocyte and neutrophil counts were associated with higher risk of incident CVD. While RBC count and haematocrit showed no clear association, individuals with greater MCV and RDW were at higher risk of CVD. Platelets were only associated with CVD risk for count values above the clinically defined normal range. However, only total and subtypes of WBC provide a modest improvement for the prediction of 10-year CVD risk when information on traditional CVD risk factors is available.

### RBC

Only one study in the general population reported weak associations between RBC count and CVD risk, while no association was found with risk of CHD^8^. These findings differ from the present study, where no association was observed neither with CVD nor CHD. A novel result of the present study is a higher risk of stroke but not of CHD among those with clinically defined low levels of RBC. In contrast, haematocrit has been the focus of various studies. A meta-analysis of prospective studies in healthy populations concluded that elevated haematocrit was a weak risk factor for CHD^9^. These results were not supported here in relation to CHD, except a weak association with CVD after excluding prevalent cases of cancer and a stronger association in current smokers only. A more recent analysis^31^ showed no association between haematocrit and CVD risk after adjustment for CVD risk factors, in line with our results. We did not find an association between MCV and CHD, similarly to a myocardial infarction case-control study^32^, but we uncovered a novel finding of an association between elevated MCV and CVD risk, driven by a strong association with risk of stroke. A handful of studies reported associations between RDW and CVD in general populations^12–15,33,34^, with four stemming from two cohorts (the Tromsø Study and the Malmö Diet and Cancer Study). Elevated RDW was associated with higher risk of total CVD in Israel^12^, and higher risk of stroke in the Malmö^34^ and Tromsø^14^ studies, but not in Taiwan^33^, while RDW was weakly or non-significantly associated with the risk of CHD^13,15,33^. As in the Taiwan study, we did not find an association with stroke risk nor CHD. A meta-analysis of our findings with existing studies show an overall significant positive association with total CVD (combined HR = 1.20 (1.08, 1.34), CHD (1.19 [1.08, 1.31]) and stroke (1.30 [1.15, 1.46]) (Supplemental Fig. 1). Hence, our findings add to the emerging evidence of RDW as a risk factor for CVD.

RDW has been shown to be associated with inflammation^35^ and it may increase in response to pro-inflammatory cytokines. Those cytokines can interact with erythropoietin in the bone marrow, leading to a lower production of RBC. Cytokines can also act as suppressors of RBC maturation leading to an increased number of immature RBC, which may reflect higher RDW levels^36^. Oxidative stress, poor nutrition, hypertension and dyslipidemia are factors that can cause a RDW increase, justifying that this inexpensive measure can be used as a predictor of CVD^37^.

### WBC

Our results of strong linear associations adjusted for multiple known CVD risk factors between increased prediagnostic WBC and CVD risk complement a long-existing body of literature^2,4,38^, confirming the relationship is “strong, consistent, dose-dependent, independent, biologically plausible”^3^. However, we also observed independent associations for differential counts, namely lymphocyte, monocyte and neutrophil counts with CVD risk, whereas the existing studies have yielded mixed results. A meta-analysis in 2004^4^ found only neutrophils to be associated with CHD, which was confirmed in subsequent studies^38,39^. A recent UK large cohort study did not find any association of eosinophils or lymphocytes with stroke or CHD^40^. Our results for stroke are concordant with the literature^41,42^ as neutrophils were the only component of the WBC count associated with stroke but we did not detect a significant association between neutrophils and CHD. When adjusting for known CVD risk factors, the association between the neutrophil and risk of stroke disappeared, indicating that the effect of neutrophils is not independent from those CVD risk factors. Our results on CVD risk provide a new insight on the usefulness of the differential WBC counts as predictors, but the relative elevation of the WBC subtypes (adjusting for total WBC count) was not associated with CVD risk.

### Platelets

Only few population-based studies examined associations with platelet count^19,43,44^. One cohort study reported an increased risk of CHD mortality in men in the top quartile of platelet counts^43^, while another observed an increased risk of incident CVD for platelet counts >300 vs 200–250 10^9^/L^19^, in line with our results of an elevated CVD and CHD risk in the high (>400) vs normal (150–400) range. A U-shape was described with stroke risk in the Caerphilly Prospective Study^44^, but these results were not replicated in our study. Despite an absence of associations with the plateletcrit in the total population, our finding that plateletcrit was positively associated with CVD in never smokers, might still support a role of this characteristic in the aetiology of CVD. The MPV, which is related to platelet activity (larger platelets are metabolically and enzymatically more active and have greater prothrombotic potential), have not been consistently associated with CHD in previously healthy patients, but more consistently so with stroke^45^. It seems to be a more useful prognostic biomarker in patients with pre-existing cardiovascular disease. Accordingly, there was no association between MPV and risk of CVD in our population, neither with risks of stroke and CHD. One study investigated the role of PDW^46^ and showed no association with CHD. The absence of association in our study was however modified by sex: in men, higher PDW was associated with higher CVD and CHD risk, whereas women displayed the opposite trend. A role of sex hormones on PDW has been shown in women, as postmenopausal women (lower estradiol levels) had less platelet activation than premenopausal women, whereas hormone replacement therapy can increase MPV and platelet activity^47^. We hypothesised that the association between PDW and CHD risk in women may be driven by the protective effect of HRT on CHD risk. However, when use of HRT and menopausal status were taken into account, the strong inverse association remained. The mechanisms underlying this gender difference are yet to be determined. The positive associations of PDW with CVD and CHD in men are strong and are the first to be reported in a previously healthy population.

### 10-year CVD risk prediction

Promising biomarkers associated with CVD, including lipid, inflammatory and genetic have failed to show a strong incremental value to established risk prediction models^48^. The increase in C-statistic beyond the SCORE model observed with lymphocytes (+0.0024) is of the same magnitude as the increase observed with the addition of C-reactive protein in a meta-analysis of 52 prospective studies (+0.0039)^49^. Together with the positive IDI for counts of total and subtypes of WBC, our results suggest that WBC count provided by simple complete blood count test may me as useful as a CRP testing to help identifying individuals at risk of future CVD.

### Limitations

Our study has limitations. Firstly, the multiple comparisons inflate the probability of type I error and of chance findings. Secondly, we rely on a single measurement of the complete blood count, which may be affected by short-term physiological changes, such as an acute infection. Finally, despite careful attention given to the adjustment of the models, residual confounding may occur because of measurement error or unmeasured risk factors, such as specific medications likely to influence some blood count parameters. The main strength of our study is its large sample size, drawn from the general population, with a broad age range and a long follow-up. Blood samples were collected at baseline and measured with standardised tests, allowing comparability with other studies. The quality of the outcome ascertainment is high as it was determined through linkage with hospital records and death registry.

## Conclusions

In this population-based prospective cohort study with a long-term follow-up, we were able to confirm the strong association between WBC, lymphocyte, monocyte and neutrophil counts and CVD risk. However, caution is warranted as no associations was found between WBC counts and CVD in never and former smokers. We also uncovered associations with other elements of the blood count, namely red blood cell mean volume and distribution width, and platelet count. These inexpensive, routinely tested, widely available measures may help identify patients at risk of future CVD, but only WBC counts seem to provide a small incremental predictive value for the estimation of 10-year CVD risk, therefore several findings still warrant replication in other large prospective cohort studies.

## Electronic supplementary material

Supplementary information
