# Supplementary material for: Elements of the complete blood count associated with cardiovascular disease incidence: Findings from the EPIC-NL cohort study
Source: Sci Rep. 2018 Feb 19;8:3290. doi: 10.1038/s41598-018-21661-x (PMC5818488; doi:10.1038/s41598-018-21661-x)

**Supplemental Material**

**Elements of the complete blood count associated with cardiovascular disease incidence: Findings from the EPIC-NL cohort study**

Camille Lassale PhD^1,2*^, Alyscia Curtis MSc ^2^, Itziar Abete PhD^2,3^, Yvonne. T. van der Schouw PhD^4^, W.M.Monique Verschuren PhD^4,5^, Yunxia Lu PhD^6,7^, H.B(as). Bueno-de-Mesquita PhD^2,5,8^

1. Department of Epidemiology and Public Health, University College London, 1-19 Torrington Place, London WC1E 7HB, United Kingdom

2. Department of Epidemiology and Biostatistics, School of Public Health, Imperial College London, Norfolk Place, London W2 1PG, United Kingdom

3. Nutrition Research Center, University of Navarra, 31010 Pamplona, Spain

4. Julius Center for Health Sciences and Primary Care, University Medical Center Utrecht, Utrecht, The Netherlands.

5. Center for Nutrition, Prevention and Health Services, National Institute for Public Health and the Environment (RIVM), 3720 BA, Bilthoven, The Netherlands

6. Program in Public Health, College of Health Sciences, University of California Irvine, CA, United States

7. Department of Molecular Medicine and Surgery, Karolinska Institutet, Stockholm, Sweden

8. Department of Social & Preventive Medicine, Faculty of Medicine, University of Malaya, Pantai Valley, 50603, Kuala Lumpur, Malaysia.

**Supplemental Information**: Logistics of storage of blood samples in EPIC-NL

In the MORGEN-EPIC study, the samples were kept at room temperature for thirty minutes to three hours in the peripheral study centers, allowing slight swelling of cells and stabilization of size, after which they were stored at +5°C and transported overnight in a coolbox to the central Cryolaboratory of RIVM.

In the Prospect-EPIC study, the samples were stored at room temperature until the arrival at the central Cryolaboratory at the end of the day. During the night, the samples were stored at +5°C. The next day the samples of both studies were again kept at room temperature for at least thirty minutes and measured in a blood cell counter (Coulter counter MAXM, Coulter Electronics).

**Supplemental Table 1.** Outcome definitions according to ICD 9 (morbidity) and ICD 10 (mortality) codes for the definition of total CVD

| **ICD 9** | **Description** | **ICD 10**  **(cause of death)** | **Description** |  |
| --- | --- | --- | --- | --- |
| 410-414 | Ischemic heart diseases | I20-I25 | Ischemic heart diseases | CHD |
| 427.5 | Cardiac arrest | I46 | Cardiac arrest |  |
| 428 | Heart failure | I50 | Heart failure |  |
| 415.1 | Pulmonary embolism and infarction | I26 | Pulmonary embolism |  |
| 430-438 | Cerebrovascular disease | I60-I69 except I68 | Cerebrovascular diseases except Cerebrovascular disorders in diseases classified elsewhere | Stroke |
|  |  | G45 | Transient cerebral ischemic attacks and related syndromes |  |
| 440 | Atherosclerosis | I70 | Atherosclerosis |  |
| 441 | Aortic aneurysm and dissection | I71 | Aortic aneurysm and dissection, |  |
| 442 | Other aneurysm | I72 | Other aneurysm |  |
| 443.9 | Peripheral vascular disease, unspecified | I73 | Other peripheral vascular diseases |  |
| 444 | Arterial embolism and thrombosis | I74 | Aerterial embolism and thrombosis |  |
| 798.1, 798.2, 798.9 | Suddent death, cause unknown (except sudden infant death) | R99 | Ill-defined and unknown cause of mortality |  |

**Supplemental Table 2.** Multivariate (Model 1)* hazard ratios HRs (95% confidence interval) for cardiovascular disease, stroke and coronary heart disease associated with tertiles of elements of the complete blood count, n= 14,362, EPIC-NL

|  |  | **CVD** | | | | **Stroke** | | | **CHD** | | |
| --- | --- | --- | --- | --- | --- | --- | --- | --- | --- | --- | --- |
|  |  | **N total** | **N cases** | **HR (95 %CI)** | **P trend^a^** | **N cases** | **HR (95 %CI)** | **P trend^a^** | **N Cases** | **HR (95 %CI)** | **P trend^a^** |
| **RBC** |  | **14362** | **992** |  |  | **196** |  |  | **589** |  |  |
| RBC (10^12^ cells/L) ^b^ | T1 | 4761 | 330 | 1 (ref) | 0.44 | 65 | 1 (ref) | 0.65 | 195 | 1 (ref) | 0.69 |
|  | T2 | 4855 | 297 | 0.82 (0.69; 0.97) |  | 48 | 0.62 (0.42; 0.92) |  | 181 | 0.92 (0.74; 1.16) |  |
|  | T3 | 4746 | 365 | 0.92 (0.75; 1.12) |  | 83 | 0.83 (0.54; 1.29) |  | 213 | 1.05 (0.8; 1.36) |  |
| Haematocrit (L/L) | T1 | 4879 | 293 | 1 (ref) | 0.13 | 53 | 1 (ref) | 0.05 | 185 | 1 (ref) | 0.93 |
|  | T2 | 4646 | 311 | 1.02 (0.87; 1.20) |  | 56 | 1.06 (0.73; 1.54) |  | 183 | 0.93 (0.76; 1.15) |  |
|  | T3 | 4837 | 388 | 1.13 (0.96; 1.32) |  | 87 | 1.37 (0.96; 1.95) |  | 221 | 0.99 (0.81; 1.22) |  |
| MCV (fL) ^c^ | T1 | 4769 | 274 | 1 (ref) | 0.07 | 44 | 1 (ref) | **0.05** | 174 | 1 (ref) | 0.63 |
|  | T2 | 4797 | 336 | 1.13 (0.96; 1.32) |  | 74 | 1.56 (1.08; 2.27) |  | 194 | 0.98 (0.79; 1.20) |  |
|  | T3 | 4796 | 382 | 1.17 (0.99; 1.37) |  | 78 | 1.54 (1.04; 2.26) |  | 221 | 1.05 (0.85; 1.30) |  |
| RDW (%) ^d^ | T1 | 4409 | 234 | 1 (ref) | **0.05** | 51 | 1 (ref) | 0.63 | 136 | 1 (ref) | 0.27 |
|  | T2 | 5536 | 368 | 1.02 (0.87; 1.21) |  | 72 | 0.93 (0.65; 1.34) |  | 220 | 1.06 (0.85; 1.32) |  |
|  | T3 | 4417 | 390 | 1.17 (0.98; 1.38) |  | 73 | 1.09 (0.75; 1.57) |  | 233 | 1.13 (0.90; 1.42) |  |
| **WBC** |  |  |  |  |  |  |  |  |  |  |  |
| WBC (10^9^cells/L) ^e^ | T1 | 4740 | 247 | 1 (ref) | **<.0001** | 41 | 1 (ref) | **0.02** | 154 | 1 (ref) | **0.01** |
|  | T2 | 4779 | 290 | 1.05 (0.88; 1.25) |  | 70 | 1.61 (1.09; 2.36) |  | 167 | 0.92 (0.74; 1.16) |  |
|  | T3 | 4843 | 455 | 1.45 (1.22; 1.71) |  | 85 | 1.63 (1.09; 2.44) |  | 268 | 1.28 (1.03; 1.59) |  |
| Lymphocytes (109cells/L) | T1 | 4910 | 283 | 1 (ref) | **0.004** | 60 | 1 (ref) | 0.16 | 157 | 1 (ref) | **0.04** |
|  | T2 | 4924 | 310 | 1.04 (0.89; 1.23) |  | 58 | 1.00 (0.7; 1.44) |  | 193 | 1.14 (0.92; 1.42) |  |
|  | T3 | 4528 | 399 | 1.26 (1.07; 1.48) |  | 78 | 1.27 (0.89; 1.82) |  | 239 | 1.26 (1.01; 1.57) |  |
| Monocyte (109cells/L) | T1 | 4586 | 274 | 1 (ref) | **0.001** | 54 | 1 (ref) | **0.03** | 159 | 1 (ref) | 0.06 |
|  | T2 | 4537 | 298 | 1.03 (0.87; 1.21) |  | 54 | 0.99 (0.68; 1.43) |  | 188 | 1.03 (0.83; 1.28) |  |
|  | T3 | 5239 | 420 | 1.25 (1.07; 1.47) |  | 88 | 1.28 (0.91; 1.81) |  | 242 | 1.18 (0.96; 1.46) |  |
| Neutrophils (109cells/L) | T1 | 4626 | 235 | 1 (ref) | **0.0003** | 41 | 1 (ref) | **0.03** | 153 | 1 (ref) | 0.10 |
|  | T2 | 4982 | 339 | 1.16 (0.98; 1.37) |  | 70 | 1.41 (0.96; 2.07) |  | 194 | 1.01 (0.81; 1.26) |  |
|  | T3 | 4754 | 418 | 1.36 (1.15; 1.62) |  | 85 | 1.60 (1.08; 2.36) |  | 242 | 1.18 (0.95; 1.47) |  |
| **Platelet** |  |  |  |  |  |  |  |  |  |  |  |
| Platelet (10^9^cells/L) | T1 | 4769 | 332 | 1 (ref) | 0.30 | 63 | 1 (ref) | 0.21 | 194 | 1 (ref) | 0.62 |
|  | T2 | 4808 | 305 | 0.92 (0.79; 1.08) |  | 59 | 0.97 (0.68; 1.38) |  | 183 | 0.95 (0.77; 1.16) |  |
|  | T3 | 4785 | 355 | 1.07 (0.92; 1.25) |  | 74 | 1.24 (0.89; 1.73) |  | 212 | 1.04 (0.85; 1.27) |  |
| Plateletcrit (L/L) | T1 | 4756 | 303 | 1 (ref) | 0.02 | 61 | 1 (ref) | 0.17 | 180 | 1 (ref) | 0.30 |
|  | T2 | 4789 | 310 | 1.00 (0.85; 1.17) |  | 57 | 0.96 (0.67; 1.38) |  | 190 | 1.03 (0.84; 1.27) |  |
|  | T3 | 4817 | 379 | 1.19 (1.02; 1.38) |  | 78 | 1.27 (0.91; 1.77) |  | 219 | 1.11 (0.90; 1.35) |  |
| MPV (fL) | T1 | 5022 | 337 | 1 (ref) | 0.85 | 71 | 1 (ref) | 0.22 | 200 | 1 (ref) | 0.83 |
|  | T2 | 4369 | 293 | 0.95 (0.81; 1.11) |  | 61 | 0.97 (0.69; 1.36) |  | 179 | 1.00 (0.82; 1.23) |  |
|  | T3 | 4971 | 362 | 0.98 (0.84; 1.14) |  | 64 | 0.81 (0.58; 1.14) |  | 210 | 0.98 (0.80; 1.20) |  |
| PDW (%) ^f^ | T1 | 4599 | 290 | 1 (ref) | 0.69 | 52 | 1 (ref) | 0.47 | 182 | 1 (ref) | 0.57 |
|  | T2 | 5017 | 344 | 0.98 (0.84; 1.15) |  | 68 | 1.05 (0.73; 1.51) |  | 205 | 0.93 (0.76; 1.15) |  |
|  | T3 | 4746 | 358 | 1.01 (0.86; 1.19) |  | 76 | 1.11 (0.77; 1.61) |  | 202 | 0.91 (0.73; 1.12) |  |

*Model adjusted for age, smoking status and intensity (7 categories), body mass index (BMI, continuous), Waist-to-hip ratio (WHR, continuous), physical activity level (Cambridge index, 4 categories), educational level (low, medium, high), alcohol intake (non drinker, occasional drinker, frequent drinker). Stratified by sex and cohort center. Age is the underlying time variable. Exit age is age at first outcome of interest or censoring.

^a^ p-value for trend calculated modelling the median of each parameter in each tertile as a continuous variable

^b^ Red blood cells: further adjusted for haemoglobin; ^c^ Mean corpuscular volume; ^d^ Red cell distribution width: further adjusted for haemoglobin, RBC, WBC and platelet count; ^e^ White blood cells; ^f^ Platelet distribution width: further adjusted for platelet count;

Abbreviations: CVD, cardiovascular disease; CHD, coronary heart disease

Cut-off points for tertiles:

Men: RBC 4.9, 5.2; Haematocrit 0.45, 0.47; MCV 89.7, 92.7; RDW 12, 12.4; WBC 5.7, 7.0; Lymphocyte 1.7, 2.1; Monocyte 0.5, 0.6; Neutrophils 3.1, 4.1; Platelets 212, 253; Plateletcrit 0.19, 0.23; MPV 8.6, 9.3; PDW 15.6, 15.9;

Women: RBC 4.3, 4.6; Haematocrit 0.39,0.42; MCV 89.3, 92.5; RDW 11.9, 12.4; WBC 5.7, 7.1; Lymphocyte 1.7, 2.1; Monocyte 0.4, 0.5; Neutrophils 3.3, 4.4; Platelets 233, 279; Plateletcrit 0.21, 0.25; MPV 8.7, 9.4; PDW 15.5, 15.8;

**Supplemental Table 3.** Multivariate (Model 1)* hazard ratios HRs (95% confidence interval) for cardiovascular disease, stroke and coronary heart disease associated with tertiles of elements of the complete blood count, in participants free of cancer at baseline n= 13,820, EPIC-NL

|  |  | **CVD** | | | | **Stroke** | | | **CHD** | | |
| --- | --- | --- | --- | --- | --- | --- | --- | --- | --- | --- | --- |
|  |  | **N total** | **N cases** | **HR (95 %CI)** | **P trend ^a^** | **N cases** | **HR (95 %CI)** | **P trend** | **N Cases** | **HR (95 %CI)** | **P trend ^a^** |
| **RBC** |  |  |  |  |  |  |  |  |  |  |  |
| RBC (10^12^ cells/L) ^b^ | T1 | 4647 | 309 | 1 (ref) | 0.72 | 61 | 1 (ref) | 0.74 | 180 | 1 (ref) | 0.41 |
|  | T2 | 4580 | 278 | 0.85 (0.71; 1.01) |  | 44 | 0.62 (0.41; 0.94) |  | 168 | 0.96 (0.76; 1.21) |  |
|  | T3 | 4593 | 351 | 0.96 (0.78; 1.18) |  | 81 | 0.90 (0.57; 1.42) |  | 204 | 1.11 (0.85; 1.46) |  |
| Haematocrit (L/L) | T1 | 4671 | 268 | 1 (ref) | **0.04** | 47 | 1 (ref) | **0.01** | 168 | 1 (ref) | 0.65 |
|  | T2 | 4540 | 305 | 1.08 (0.92; 1.28) |  | 54 | 1.11 (0.75; 1.64) |  | 176 | 0.96 (0.78; 1.20) |  |
|  | T3 | 4609 | 365 | **1.19 (1.01; 1.40)** |  | 85 | **1.59 (1.1; 2.3)** |  | 208 | 1.05 (0.85; 1.30) |  |
| MCV (fL) ^c^ | T1 | 4602 | 259 | 1 (ref) | 0.07 | 43 | 1 (ref) | 0.06 | 163 | 1 (ref) | 0.60 |
|  | T2 | 4623 | 320 | 1.14 (0.96; 1.34) |  | 69 | 1.51 (1.03; 2.22) |  | 184 | 0.99 (0.80; 1.23) |  |
|  | T3 | 4595 | 359 | 1.17 (0.99; 1.39) |  | 74 | 1.47 (0.99; 2.18) |  | 205 | 1.06 (0.85; 1.32) |  |
| RDW (%) ^d^ | T1 | 4268 | 224 | 1 (ref) | 0.09 | 49 | 1 (ref) | 0.62 | 131 | 1 (ref) | 0.40 |
|  | T2 | 4797 | 313 | 1.03 (0.86; 1.22) |  | 60 | 0.93 (0.64; 1.36) |  | 186 | 1.05 (0.83; 1.32) |  |
|  | T3 | 4755 | 401 | 1.15 (0.97; 1.36) |  | 77 | 1.07 (0.74; 1.56) |  | 235 | 1.10 (0.88; 1.39) |  |
| **WBC** |  |  |  |  |  |  |  |  |  |  |  |
| WBC (10^9^cells/L) ^e^ | T1 | 4530 | 233 | 1 (ref) | **<.0001** | 39 | 1 (ref) | **0.02** | 146 | 1 (ref) | **0.02** |
|  | T2 | 4667 | 281 | 1.06 (0.89; 1.26) |  | 67 | 1.58 (1.06; 2.35) |  | 159 | 0.91 (0.72; 1.15) |  |
|  | T3 | 4623 | 424 | **1.45 (1.21; 1.72)** |  | 80 | **1.69 (1.11; 2.55)** |  | 247 | **1.27 (1.01; 1.59)** |  |
| Lymphocytes count | T1 | 4685 | 267 | 1 (ref) | **0.01** | 58 | 1 (ref) | 0.24 | 145 | 1 (ref) | **0.04** |
|  | T2 | 4755 | 293 | 1.03 (0.87; 1.22) |  | 55 | 0.96 (0.66; 1.40) |  | 182 | 1.15 (0.92; 1.44) |  |
|  | T3 | 4380 | 378 | **1.25 (1.06; 1.48)** |  | 73 | 1.23 (0.85; 1.79) |  | 225 | **1.28 (1.02; 1.60)** |  |
| Monocyte count | T1 | 4399 | 261 | 1 (ref) | **0.001** | 54 | 1 (ref) | 0.08 | 150 | 1 (ref) | 0.06 |
|  | T2 | 4378 | 284 | 1.02 (0.86; 1.21) |  | 51 | 0.96 (0.65; 1.42) |  | 177 | 1.02 (0.82; 1.28) |  |
|  | T3 | 5043 | 393 | **1.24 (1.05; 1.45)** |  | 81 | 1.25 (0.87; 1.79) |  | 225 | 1.18 (0.95; 1.47) |  |
| Neutrophils count | T1 | 4430 | 221 | 1 (ref) | **0.001** | 39 | 1 (ref) | **0.03** | 144 | 1 (ref) | 0.11 |
|  | T2 | 4791 | 321 | 1.17 (0.98; 1.39) |  | 66 | 1.39 (0.93; 2.07) |  | 181 | 1.01 (0.81; 1.26) |  |
|  | T3 | 4599 | 396 | **1.37 (1.15; 1.63)** |  | 81 | **1.62 (1.08; 2.42)** |  | 227 | 1.18 (0.94; 1.48) |  |
| **Platelet** |  |  |  |  |  |  |  |  |  |  |  |
| Platelet (10^9^cells/L) | T1 | 4578 | 310 | 1 (ref) | 0.24 | 59 | 1 (ref) | 0.23 | 179 | 1 (ref) | 0.46 |
|  | T2 | 4618 | 290 | 0.95 (0.81; 1.11) |  | 57 | 1.01 (0.7; 1.45) |  | 171 | 0.96 (0.78; 1.19) |  |
|  | T3 | 4624 | 338 | 1.09 (0.93; 1.27) |  | 70 | 1.26 (0.89; 1.78) |  | 202 | 1.07 (0.87; 1.31) |  |
| Plateletcrit (L/L) | T1 | 4634 | 289 | 1 (ref) | **0.02** | 58 | 1 (ref) | 0.20 | 168 | 1 (ref) | 0.16 |
|  | T2 | 4608 | 293 | 1.01 (0.86; 1.19) |  | 56 | 0.99 (0.68; 1.43) |  | 176 | 1.03 (0.83; 1.28) |  |
|  | T3 | 4578 | 356 | **1.20 (1.02; 1.40)** |  | 72 | 1.26 (0.89; 1.79) |  | 208 | 1.15 (0.93; 1.42) |  |
| MPV (fL) | T1 | 4825 | 320 | 1 (ref) | 0.90 | 66 | 1 (ref) | 0.43 | 189 | 1 (ref) | 0.84 |
|  | T2 | 4188 | 272 | 0.94 (0.80; 1.10) |  | 56 | 0.92 (0.65; 1.32) |  | 163 | 0.97 (0.78; 1.20) |  |
|  | T3 | 4807 | 346 | 0.98 (0.84; 1.15) |  | 64 | 0.87 (0.61; 1.23) |  | 200 | 0.98 (0.80; 1.20) |  |
| PDW (%) ^f^ | T1 | 4460 | 276 | 1 (ref) | 0.62 | 48 | 1 (ref) | 0.26 | 174 | 1 (ref) | 0.56 |
|  | T2 | 4821 | 323 | 0.98 (0.83; 1.15) |  | 64 | 1.08 (0.74; 1.57) |  | 188 | 0.90 (0.73; 1.11) |  |
|  | T3 | 4539 | 339 | 1.02 (0.86; 1.20) |  | 74 | 1.22 (0.84; 1.79) |  | 190 | 0.90 (0.72; 1.12) |  |

*Model adjusted for age, smoking status and intensity (7 categories), body mass index (BMI, continuous), Waist-to-hip ratio (WHR, continuous), physical activity level (Cambridge index, 4 categories), educational level (low, medium, high), alcohol intake (non drinker, occasional drinker, frequent drinker). Stratified by sex and cohort center. Age is the underlying time variable. Exit age is age at first outcome of interest or censoring.

^a^ p-value for trend calculated modelling the median of each parameter in each tertile as a continuous variable

^b^ Red blood cells: further adjusted for haemoglobin; ^c^ Mean corpuscular volume; ^d^ Red cell distribution width: further adjusted for haemoglobin, RBC, WBC and platelet count; ^e^ White blood cells; ^f^ Platelet distribution width: further adjusted for platelet count;

Abbreviations: CVD, cardiovascular disease; CHD, coronary heart disease

**Supplemental Table 4.** Multivariate (Model 2 = Model 1 further adjusted for CVD risk factors)* hazard ratios HRs (95% confidence interval) for cardiovascular disease, stroke and coronary heart disease associated with tertiles of elements of the complete blood count, in participants free of cancer at baseline n= 13,820, EPIC-NL

|  |  | **CVD** | | | | **Stroke** | | | **CHD** | | |
| --- | --- | --- | --- | --- | --- | --- | --- | --- | --- | --- | --- |
|  |  | **N total** | **N cases** | **HR (95 %CI)** | **P trend ^a^** | **N cases** | **HR (95 %CI)** | **P trend** | **N Cases** | **HR (95 %CI)** | **P trend ^a^** |
| **RBC** |  |  |  |  |  |  |  |  |  |  |  |
| RBC (10^12^ cells/L) ^b^ | T1 | 4647 | 309 | 1 (ref) | 0.29 | 61 | 1 (ref) | 0.52 | 180 | 1 (ref) | 0.82 |
|  | T2 | 4580 | 278 | 0.84 (0.70; 1.00) |  | 44 | 0.62 (0.41; 0.94) |  | 168 | 0.93 (0.74; 1.18) |  |
|  | T3 | 4593 | 351 | 0.89 (0.73; 1.09) |  | 81 | 0.84 (0.54; 1.33) |  | 204 | 1.03 (0.78; 1.35) |  |
| Haematocrit (L/L) | T1 | 4671 | 268 | 1 (ref) | 0.50 | 43 | 1 (ref) | 0.10 | 163 |  | 0.64 |
|  | T2 | 4540 | 305 | 1.02 (0.87; 1.21) |  | 69 | 1.01 (0.68; 1.50) |  | 184 | 0.92 (0.74; 1.14) |  |
|  | T3 | 4609 | 365 | 1.06 (0.90; 1.25) |  | 74 | 1.33 (0.92; 1.94) |  | 205 | 0.95 (0.77; 1.18) |  |
| MCV (fL) ^c^ | T1 | 4602 | 259 | 1 (ref) | **0.01** | 47 | 1 (ref) | **0.02** | 168 | 1 (ref) | 0.32 |
|  | T2 | 4623 | 320 | 1.17 (0.99; 1.38) |  | 54 | 1.59 (1.08; 2.34) |  | 176 | 1.02 (0.82; 1.27) |  |
|  | T3 | 4595 | 359 | **1.24 (1.04; 1.47)** |  | 85 | **1.59 (1.06; 2.38)** |  | 208 | 1.11 (0.89; 1.39) |  |
| RDW (%) ^d^ | T1 | 4268 | 224 | 1 (ref) | **0.02** | 49 | 1 (ref) | 0.52 | 131 |  | 0.23 |
|  | T2 | 4797 | 313 | 1.06 (0.89; 1.26) |  | 60 | 0.97 (0.67; 1.40) |  | 186 | 1.09 (0.87; 1.37) |  |
|  | T3 | 4755 | 401 | **1.22 (1.02; 1.45)** |  | 77 | 1.12 (0.76; 1.64) |  | 235 | 1.16 (0.91; 1.46) |  |
| WBC |  |  |  |  |  |  |  |  |  |  |  |
| WBC (10^9^cells/L) ^e^ | T1 | 4530 | 233 | 1 (ref) | **0.001** | 39 | 1 (ref) | 0.12 | 146 | 1 (ref) | 0.11 |
|  | T2 | 4667 | 281 | 1.01 (0.85; 1.21) |  | 67 | 1.49 (1.00; 2.23) |  | 159 | 0.88 (0.70; 1.10) |  |
|  | T3 | 4623 | 424 | **1.31 (1.10; 1.56)** |  | 80 | 1.47 (0.97; 2.24) |  | 247 | 1.15 (0.92; 1.45) |  |
| Lymphocytes count | T1 | 4685 | 267 | 1 (ref) | **0.03** | 58 | 1 (ref) | 0.37 | 145 | 1 (ref) | 0.10 |
|  | T2 | 4755 | 293 | 1.02 (0.86; 1.21) |  | 55 | 0.94 (0.65; 1.37) |  | 182 | 1.14 (0.91; 1.42) |  |
|  | T3 | 4380 | 378 | **1.19 (1.00; 1.41)** |  | 73 | 1.16 (0.80; 1.69) |  | 225 | 1.21 (0.97; 1.52) |  |
| Monocyte count | T1 | 4399 | 261 | 1 (ref) | **0.008** | 54 | 1 (ref) | 0.19 | 150 | 1 (ref) | 0.12 |
|  | T2 | 4378 | 284 | 1.01 (0.86; 1.20) |  | 51 | 0.95 (0.65; 1.40) |  | 177 | 1.02 (0.82; 1.27) |  |
|  | T3 | 5043 | 393 | **1.19 (1.01; 1.39)** |  | 81 | 1.16 (0.81; 1.65) |  | 225 | 1.14 (0.92; 1.42) |  |
| Neutrophils count | T1 | 4430 | 221 | 1 (ref) | **0.02** | 39 | 1 (ref) | 0.17 | 144 | 1 (ref) | 0.43 |
|  | T2 | 4791 | 321 | 1.14 (0.96; 1.36) |  | 66 | 1.34 (0.90; 2.00) |  | 181 | 0.99 (0.79; 1.24) |  |
|  | T3 | 4599 | 396 | **1.24 (1.04; 1.48)** |  | 81 | 1.39 (0.93; 2.09) |  | 227 | 1.08 (0.86; 1.36) |  |
| Platelet |  |  |  |  |  |  |  |  |  |  |  |
| Platelet (10^9^cells/L) | T1 | 4578 | 310 | 1 (ref) | 0.38 | 59 | 1 (ref) | 0.32 | 179 | 1 (ref) | 0.57 |
|  | T2 | 4618 | 290 | 0.94 (0.80; 1.10) |  | 57 | 0.99 (0.68; 1.42) |  | 171 | 0.96 (0.77; 1.18) |  |
|  | T3 | 4624 | 338 | 1.07 (0.91; 1.25) |  | 70 | 1.22 (0.86; 1.73) |  | 202 | 1.05 (0.85; 1.29) |  |
| Plateletcrit (L/L) | T1 | 4634 | 289 | 1 (ref) | **0.05** | 58 | 1 (ref) | 0.30 | 168 | 1 (ref) | 0.32 |
|  | T2 | 4608 | 293 | 1.00 (0.85; 1.18) |  | 56 | 0.98 (0.68; 1.43) |  | 176 | 1.04 (0.84; 1.29) |  |
|  | T3 | 4578 | 356 | 1.16 (0.99; 1.36) |  | 72 | 1.21 (0.86; 1.71) |  | 208 | 1.11 (0.90; 1.36) |  |
| MPV (fL) | T1 | 4825 | 320 | 1 (ref) | 0.63 | 66 | 1 (ref) | 0.57 | 189 | 1 (ref) | 0.20 |
|  | T2 | 4188 | 272 | 0.93 (0.79; 1.09) |  | 56 | 1.02 (0.70; 1.49) |  | 163 | 0.86 (0.70; 1.06) |  |
|  | T3 | 4807 | 346 | 0.93 (0.79; 1.10) |  | 64 | 1.10 (0.75; 1.61) |  | 200 | 0.83 (0.66; 1.03) |  |
| PDW (%) ^f^ | T1 | 4460 | 276 | 1 (ref) | 0.80 | 48 | 1 (ref) | 0.38 | 174 | 1 (ref) | 0.74 |
|  | T2 | 4821 | 323 | 0.94 (0.80; 1.10) |  | 64 | 0.93 (0.65; 1.33) |  | 188 | 0.97 (0.78; 1.20) |  |
|  | T3 | 4539 | 339 | 0.97 (0.84; 1.13) |  | 74 | 0.85 (0.60; 1.21) |  | 190 | 0.96 (0.79; 1.18) |  |

*Model adjusted for age, smoking status and intensity (7 categories), body mass index (BMI, continuous), Waist-to-hip ratio (WHR, continuous), physical activity level (Cambridge index, 4 categories), educational level (low, medium, high), alcohol intake (non drinker, occasional drinker, frequent drinker), systolic blood pressure, HDL cholesterol and diabetes. Stratified by sex and cohort center. Age is the underlying time variable. Exit age is age at first outcome of interest or censoring.

^a^ p-value for trend calculated modelling the median of each parameter in each tertile as a continuous variable

^b^ Red blood cells: further adjusted for haemoglobin; ^c^ Mean corpuscular volume; ^d^ Red cell distribution width: further adjusted for haemoglobin, RBC, WBC and platelet count; ^e^ White blood cells; ^f^ Platelet distribution width: further adjusted for platelet count;

Abbreviations: CVD, cardiovascular disease; CHD, coronary heart disease

**Supplemental Table 5.** Multivariate * hazard ratios HRs (95% confidence interval) for cardiovascular disease, stroke and coronary heart disease associated with tertiles of white blood cell subtypes adjusted for total WBC count, n= 14,362, EPIC-NL

|  |  | **CVD** | | | | **Stroke** | | | **CHD** | | |
| --- | --- | --- | --- | --- | --- | --- | --- | --- | --- | --- | --- |
|  |  | **N total** | **N cases** | **HR (95 %CI)** | **P trend^a^** | **N cases** | **HR (95 %CI)** | **P trend^a^** | **N Cases** | **HR (95 %CI)** | **P trend^a^** |
|  |  | **14362** | **992** |  |  | **196** |  |  | **589** |  |  |
| Lymphocytes (10^9^cells/L) | T1 | 4910 | 283 | 1 (ref) | 0.52 | 60 | 1 (ref) | 0.79 | 157 | 1 (ref) | 0.41 |
|  | T2 | 4924 | 310 | 0.98 (0.83;1.16) |  | 58 | 0.92 (0.63; 1.33) |  | 193 | 1.10 (0.88; 1.37) |  |
|  | T3 | 4528 | 399 | 1.05 (0.88;1.26) |  | 78 | 1.04 (0.70; 1.57) |  | 239 | 1.11 (0.87; 1.42) |  |
| Monocyte (10^9^cells/L) | T1 | 4586 | 274 | 1 (ref) | 0.09 | 54 | 1 (ref) | 0.26 | 159 | 1 (ref) | 0.31 |
|  | T2 | 4537 | 298 | 1.01 (0.85;1.19) |  | 54 | 1.00 (0.68; 1.46) |  | 188 | 1.01 (0.82; 1.26) |  |
|  | T3 | 5239 | 420 | 1.11 (0.94;1.31) |  | 88 | 1.15 (0.80; 1.66) |  | 242 | 1.09 (0.87; 1.35) |  |
| Neutrophils (10^9^cells/L) | T1 | 4626 | 235 | 1 (ref) | 0.63 | 41 | 1 (ref) | 0.92 | 153 | 1 (ref) | 0.45 |
|  | T2 | 4982 | 339 | 1.03 (0.86;1.23) |  | 70 | 1.21 (0.80; 1.82) |  | 194 | 0.92 (0.73; 1.16) |  |
|  | T3 | 4754 | 418 | 0.96 (0.76;1.22) |  | 85 | 1.04 (0.61; 1.79) |  | 242 | 0.88 (0.65; 1.20) |  |

*Model adjusted for age, smoking status and intensity (7 categories), body mass index (BMI, continuous), Waist-to-hip ratio (WHR, continuous), physical activity level (Cambridge index, 4 categories), educational level (low, medium, high), alcohol intake (non drinker, occasional drinker, frequent drinker), systolic blood pressure, HDL cholesterol and diabetes. Stratified by sex and cohort center. Age is the underlying time variable. Exit age is age at first outcome of interest or censoring.

**Supplemental Table 6.** Multivariate (Model 1)* hazard ratios HRs (95% confidence interval) for cardiovascular disease, stroke and coronary heart disease associated with tertiles of elements of the complete blood count, after exclusion of first 2 years of follow-up n= 14,182, EPIC-NL

|  | **CVD** | | | | **Stroke** | | | **CHD** | | |
| --- | --- | --- | --- | --- | --- | --- | --- | --- | --- | --- |
|  | **N total** | **N cases** | **HR (95 %CI)** | **P trend ^a^** | **N cases** | **HR (95 %CI)** | **P trend** | **N Cases** | **HR (95 %CI)** | **P trend ^a^** |
| **RBC** |  |  |  |  |  |  |  |  |  |  |
| RBC (10^12^ cells/L) ^b^ | 4689 | 286 | 1 (ref) | 0.38 | 56 | 1 (ref) | 0.40 | 162 | **1 (ref)** | 0.61 |
|  | 4809 | 267 | 0.83 (0.69; 1.00) |  | 41 | 0.57 (0.37; 0.88) |  | 162 | 0.98 (0.77; 1.25) |  |
|  | 4684 | 323 | 0.90 (0.73; 1.11) |  | 73 | 0.78 (0.48; 1.26) |  | 183 | 1.07 (0.80; 1.42) |  |
| Haematocrit (L/L) | 4809 | 250 | 1 (ref) | 0.06 | 45 | 1 (ref) | **0.02** | 149 | 1 (ref) | 0.48 |
|  | 4657 | 286 | 1.09 (0.92; 1.29) |  | 48 | 1.04 (0.69; 1.56) |  | 166 | 1.02 (0.82; 1.28) |  |
|  | 4716 | 340 | **1.18 (1; 1.4)** |  | 77 | **1.53 (1.05; 2.24)** |  | 192 | 1.09 (0.87; 1.36) |  |
| MCV (fL) ^c^ | 4721 | 246 | 1 (ref) | 0.10 | 40 | 1 (ref) | **0.04** | 152 | 1 (ref) | 0.79 |
|  | 4734 | 295 | 1.11 (0.94; 1.32) |  | 60 | 1.44 (0.96; 2.15) |  | 167 | 0.98 (0.79; 1.23) |  |
|  | 4727 | 335 | 1.16 (0.97; 1.38) |  | 70 | **1.56 (1.03; 2.35)** |  | 188 | 1.03 (0.82; 1.29) |  |
| RDW (%) ^d^ | 4365 | 213 | 1 (ref) | 0.07 | 46 | 1 (ref) | 0.65 | 123 | 1 (ref) | 0.21 |
|  | 4924 | 285 | 0.98 (0.82; 1.17) |  | 54 | 0.90 (0.60; 1.33) |  | 164 | 0.98 (0.77; 1.25) |  |
|  | 4893 | 378 | 1.15 (0.97; 1.37) |  | 70 | 1.07 (0.72; 1.57) |  | 220 | 1.14 (0.89; 1.44) |  |
| **WBC** |  |  |  |  |  |  |  |  |  |  |
| WBC (10^9^cells/L) ^e^ | 4693 | 226 | 1 (ref) | **0.0002** | 36 | 1 (ref) | 0.09 | 139 | 1 (ref) | 0.08 |
|  | 4732 | 260 | 1.04 (0.87; 1.25) |  | 66 | 1.73 (1.15; 2.62) |  | 144 | 0.89 (0.70; 1.13) |  |
|  | 4757 | 390 | **1.38 (1.15; 1.65)** |  | 68 | 1.59 (1.02; 2.45) |  | 224 | 1.19 (0.94; 1.51) |  |
| Lymphocytes (109cells/L) | 4869 | 258 | 1 (ref) | **0.02** | 53 | 1 (ref) | 0.32 | 140 | 1 (ref) | 0.10 |
|  | 4854 | 271 | 1.01 (0.85; 1.20) |  | 54 | 1.06 (0.72; 1.55) |  | 161 | 1.06 (0.84; 1.34) |  |
|  | 4459 | 347 | **1.23 (1.03; 1.46)** |  | 63 | 1.22 (0.82; 1.81) |  | 206 | 1.21 (0.96; 1.53) |  |
| Monocyte (109cells/L) | 4538 | 250 | 1 (ref) | **0.001** | 48 | 1 (ref) | **0.02** | 144 | 1 (ref) | 0.13 |
|  | 4473 | 254 | 0.97 (0.81; 1.15) |  | 45 | 0.97 (0.64; 1.47) |  | 154 | 0.94 (0.74; 1.19) |  |
|  | 5171 | 372 | **1.24 (1.05; 1.47)** |  | 77 | 1.41 (0.97; 2.05) |  | 209 | 1.14 (0.91; 1.42) |  |
| Neutrophils (109cells/L) | 4575 | 212 | 1 (ref) | **0.003** | 37 | 1 (ref) | 0.09 | 135 | 1 (ref) | 0.25 |
|  | 4930 | 303 | 1.16 (0.97; 1.39) |  | 63 | 1.41 (0.94; 2.12) |  | 169 | 1.03 (0.82; 1.30) |  |
|  | 4677 | 361 | **1.33 (1.11; 1.59)** |  | 70 | 1.52 (0.99; 2.31) |  | 203 | 1.14 (0.90; 1.45) |  |
| **Platelet** |  |  |  |  |  |  |  |  |  |  |
| Platelet (10^9^cells/L) | 4709 | 292 | 1 (ref) | 0.23 | 49 | 1 (ref) | **0.03** | 167 | 1 (ref) | 0.83 |
|  | 4751 | 267 | 0.92 (0.78; 1.08) |  | 51 | 1.08 (0.73; 1.60) |  | 159 | 0.96 (0.77; 1.19) |  |
|  | 4722 | 317 | 1.09 (0.93; 1.28) |  | 70 | **1.52 (1.05; 2.20)** |  | 181 | 1.01 (0.81; 1.26) |  |
| Plateletcrit (L/L) | 4707 | 269 | 1 (ref) | **0.03** | 49 | 1 (ref) | 0.07 | 157 | 1 (ref) | 0.45 |
|  | 4720 | 270 | 0.97 (0.82; 1.15) |  | 51 | 1.04 (0.70; 1.55) |  | 160 | 0.98 (0.79; 1.23) |  |
|  | 4755 | 337 | **1.18 (1.00; 1.38)** |  | 70 | 1.41 (0.97; 2.03) |  | 190 | 1.08 (0.87; 1.34) |  |
| MPV (fL) | 4951 | 292 | 1 (ref) | 0.83 | 58 | 1 (ref) | 0.29 | 171 | 1 (ref) | 0.89 |
|  | 4316 | 264 | 0.98 (0.83; 1.16) |  | 58 | 1.07 (0.75; 1.55) |  | 153 | 0.99 (0.79; 1.24) |  |
|  | 4915 | 320 | 0.98 (0.84; 1.15) |  | 54 | 0.83 (0.57; 1.20) |  | 183 | 0.98 (0.80; 1.22) |  |
| PDW (%) ^f^ | 4539 | 254 | 1 (ref) | 0.66 | 46 | 1 (ref) | 0.51 | 157 | 1 (ref) | 0.62 |
|  | 4958 | 308 | 1.01 (0.85; 1.19) |  | 61 | 1.08 (0.73; 1.59) |  | 176 | 0.94 (0.75; 1.17) |  |
|  | 4685 | 314 | 1.02 (0.86; 1.21) |  | 63 | 1.14 (0.77; 1.70) |  | 174 | 0.91 (0.72; 1.14) |  |

*Model adjusted for age, smoking status and intensity (7 categories), body mass index (BMI, continuous), Waist-to-hip ratio (WHR, continuous), physical activity level (Cambridge index, 4 categories), educational level (low, medium, high), alcohol intake (non drinker, occasional drinker, frequent drinker). Stratified by sex and cohort center. Age is the underlying time variable. Exit age is age at first outcome of interest or censoring.

^a^ p-value for trend calculated modelling the median of each parameter in each tertile as a continuous variable

^b^ Red blood cells: further adjusted for haemoglobin; ^c^ Mean corpuscular volume; ^d^ Red cell distribution width: further adjusted for haemoglobin, RBC, WBC and platelet count; ^e^ White blood cells; ^f^ Platelet distribution width: further adjusted for platelet count;

Abbreviations: CVD, cardiovascular disease; CHD, coronary heart disease

**Supplemental Table 7.** Multivariate (Model 2)* hazard ratios HRs (95% confidence interval) for cardiovascular disease, stroke and coronary heart disease associated with tertiles of elements of the complete blood count, after exclusion of first 2 years of follow-up n= 14,182, EPIC-NL

|  | **CVD** | | | | **Stroke** | | | **CHD** | | |
| --- | --- | --- | --- | --- | --- | --- | --- | --- | --- | --- |
|  | **N total** | **N cases** | **HR (95 %CI)** | **P trend ^a^** | **N cases** | **HR (95 %CI)** | **P trend** | **N Cases** | **HR (95 %CI)** | **P trend^a^** |
| **RBC** |  |  |  |  |  |  |  |  |  |  |
| RBC (10^12^ cells/L) ^b^ | 4689 | 286 | 1 (ref) | 0.12 | 56 | 1 (ref) | 0.24 | 162 | 1 (ref) | 0.96 |
|  | 4809 | 267 | 0.82 (0.68; 0.98) |  | 41 | 0.57 (0.37; 0.88) |  | 162 | 0.95 (0.75; 1.21) |  |
|  | 4684 | 323 | 0.84 (0.68; 1.04) |  | 73 | 0.72 (0.45; 1.17) |  | 183 | 0.98 (0.74; 1.31) |  |
| Haematocrit (L/L) | 4809 | 250 | 1 (ref) | 0.52 | 45 | 1 (ref) | 0.16 | 149 | 1 (ref) | 0.91 |
|  | 4657 | 286 | 1.04 (0.87; 1.23) |  | 48 | 0.95 (0.63; 1.44) |  | 166 | 0.98 (0.78; 1.23) |  |
|  | 4716 | 340 | 1.06 (0.89; 1.26) |  | 77 | 1.29 (0.88; 1.89) |  | 192 | 0.99 (0.79; 1.24) |  |
| MCV (fL) ^c^ | 4721 | 246 | 1 (ref) | **0.02** | 40 | 1 (ref) | **0.01** | 152 | 1 (ref) | 0.47 |
|  | 4734 | 295 | 1.14 (0.96; 1.35) |  | 60 | **1.52 (1.01; 2.27)** |  | 167 | 1.01 (0.80; 1.26) |  |
|  | 4727 | 335 | **1.23 (1.03; 1.46)** |  | 70 | **1.71 (1.13; 2.60)** |  | 188 | 1.08 (0.86; 1.37) |  |
| RDW (%) ^d^ | 4365 | 213 | 1 (ref) | **0.01** | 46 | 1 (ref) | 0.62 | 123 | 1 (ref) | 0.10 |
|  | 4924 | 285 | 1.01 (0.85; 1.20) |  | 54 | 0.95 (0.65; 1.40) |  | 164 | 1.02 (0.81; 1.29) |  |
|  | 4893 | 378 | **1.23 (1.03; 1.47)** |  | 70 | 1.10 (0.74; 1.63) |  | 220 | 1.20 (0.94; 1.53) |  |
| **WBC** |  |  |  |  |  |  |  |  |  |  |
| WBC (10^9^cells/L) ^e^ | 4693 | 226 | 1 (ref) | **0.01** | 36 | 1 (ref) | 0.31 | 139 | 1 (ref) | 0.33 |
|  | 4732 | 260 | 1.00 (0.83; 1.19) |  | 66 | **1.62 (1.08; 2.45)** |  | 144 | 0.85 (0.67; 1.08) |  |
|  | 4757 | 390 | **1.25 (1.04; 1.49)** |  | 68 | 1.37 (0.88; 2.13) |  | 224 | 1.08 (0.86; 1.38) |  |
| Lymphocytes (109cells/L) | 4869 | 258 | 1 (ref) | 0.07 | 53 | 1 (ref) | 0.48 | 140 | 1 (ref) | 0.23 |
|  | 4854 | 271 | 0.99 (0.84; 1.18) |  | 54 | 1.03 (0.70; 1.52) |  | 161 | 1.05 (0.83; 1.32) |  |
|  | 4459 | 347 | 1.16 (0.98; 1.38) |  | 63 | 1.15 (0.77; 1.70) |  | 206 | 1.15 (0.91; 1.45) |  |
| Monocyte (109cells/L) | 4538 | 250 | 1 (ref) | **0.01** | 48 | 1 (ref) | **0.04** | 144 | 1 (ref) | 0.20 |
|  | 4473 | 254 | 0.96 (0.81; 1.15) |  | 45 | 0.96 (0.64; 1.45) |  | 154 | 0.94 (0.74; 1.18) |  |
|  | 5171 | 372 | **1.20 (1.01; 1.41)** |  | 77 | 1.31 (0.90; 1.91) |  | 209 | 1.11 (0.88; 1.38) |  |
| Neutrophils (109cells/L) | 4575 | 212 | 1 (ref) | **0.05** | 37 | 1 (ref) | 0.37 | 135 | 1 (ref) | 0.67 |
|  | 4930 | 303 | 1.13 (0.95; 1.35) |  | 63 | 1.35 (0.90; 2.03) |  | 169 | 1.00 (0.80; 1.27) |  |
|  | 4677 | 361 | **1.21 (1.01; 1.45)** |  | 70 | 1.30 (0.85; 1.98) |  | 203 | 1.05 (0.83; 1.34) |  |
| **Platelet** |  |  |  |  |  |  |  |  |  |  |
| Platelet (10^9^cells/L) | 4709 | 292 | 1 (ref) | 0.34 | 49 | 1 (ref) | **0.04** | 167 | 1 (ref) | 0.90 |
|  | 4751 | 267 | 0.91 (0.77; 1.08) |  | 51 | 1.07 (0.72; 1.58) |  | 159 | 0.97 (0.78; 1.21) |  |
|  | 4722 | 317 | 1.07 (0.91; 1.26) |  | 70 | **1.48 (1.02; 2.14)** |  | 181 | 1.00 (0.81; 1.25) |  |
| Plateletcrit (L/L) | 4707 | 269 | 1 (ref) | 0.09 | 49 | 1 (ref) | 0.13 | 157 | 1 (ref) | 0.62 |
|  | 4720 | 270 | 0.97 (0.82; 1.15) |  | 51 | 1.06 (0.71; 1.57) |  | 160 | 0.98 (0.79; 1.23) |  |
|  | 4755 | 337 | 1.14 (0.97; 1.34) |  | 70 | 1.34 (0.92; 1.93) |  | 190 | 1.05 (0.84; 1.30) |  |
| MPV (fL) | 4951 | 292 | 1 (ref) | 0.75 | 58 | 1 (ref) | 0.25 | 171 | 1 (ref) | 0.80 |
|  | 4316 | 264 | 0.98 (0.83; 1.15) |  | 58 | 1.08 (0.75; 1.56) |  | 153 | 0.98 (0.79; 1.23) |  |
|  | 4915 | 320 | 0.97 (0.83; 1.14) |  | 54 | 0.82 (0.56; 1.19) |  | 183 | 0.97 (0.79; 1.20) |  |
| PDW (%) ^f^ | 4539 | 254 | 1 (ref) | 0.62 | 46 | 1 (ref) | 0.93 | 157 | 1 (ref) | 0.25 |
|  | 4958 | 308 | 0.96 (0.82; 1.14) |  | 61 | 1.02 (0.69; 1.51) |  | 176 | 0.90 (0.72; 1.12) |  |
|  | 4685 | 314 | 0.94 (0.79; 1.12) |  | 63 | 1.02 (0.68; 1.52) |  | 174 | 0.84 (0.67; 1.06) |  |

*Model adjusted for age, smoking status and intensity (7 categories), body mass index (BMI, continuous), Waist-to-hip ratio (WHR, continuous), physical activity level (Cambridge index, 4 categories), educational level (low, medium, high), alcohol intake (non drinker, occasional drinker, frequent drinker), systolic blood pressure, HDL cholesterol, diabetes. Stratified by sex and cohort center. Age is the underlying time variable. Exit age is age at first outcome of interest or censoring.

^a^ p-value for trend calculated modelling the median of each parameter in each tertile as a continuous variable

^b^ Red blood cells: further adjusted for haemoglobin; ^c^ Mean corpuscular volume; ^d^ Red cell distribution width: further adjusted for haemoglobin, RBC, WBC and platelet count; ^e^ White blood cells; ^f^ Platelet distribution width: further adjusted for platelet count;

Abbreviations: CVD, cardiovascular disease; CHD, coronary heart disease

**Supplemental Table 8.** Multivariate (Model 2)* hazard ratios HRs (95% confidence interval) for cardiovascular disease, stroke and coronary heart disease associated with cohort- (and sex-) specific tertiles of elements of the complete blood count, n= 14,182, EPIC-NL

|  | **CVD** | | | | **Stroke** | | | **CHD** | | |
| --- | --- | --- | --- | --- | --- | --- | --- | --- | --- | --- |
|  | **N total** | **N cases** | **HR (95 %CI)** | **P trend ^a^** | **N cases** | **HR (95 %CI)** | **P trend** | **N Cases** | **HR (95 %CI)** | **P trend ^a^** |
| **RBC** |  |  |  |  |  |  |  |  |  |  |
| RBC (10^12^ cells/L) ^b^ | 4772 | 310 | 1 (ref) | 0.6225 | 58 | 1 (ref) | 0.5846 | 186 | 1 (ref) | 0.9509 |
|  | 4826 | 312 | 0.87 (0.74; 1.04) |  | 55 | 0.62 (0.41; 0.92) |  | 186 | 0.92 (0.74; 1.16) |  |
|  | 4764 | 370 | 0.88 (0.72; 1.07) |  | 83 | 0.81 (0.52; 1.26) |  | 217 | 0.98 (0.75; 1.27) |  |
| Haematocrit (L/L) | 4824 | 281 | 1 (ref) | 0.0496 | 50 | 1 (ref) | 0.329 | 176 | 1 (ref) | 0.5725 |
|  | 4743 | 307 | 0.97 (0.82; 1.14) |  | 57 | 0.94 (0.64; 1.37) |  | 185 | 0.92 (0.75; 1.14) |  |
|  | 4795 | 404 | 1.04 (0.88; 1.21) |  | 89 | 1.17 (0.82; 1.67) |  | 228 | 0.94 (0.76; 1.16) |  |
| MCV (fL) ^c^ | 4808 | 276 | 1 (ref) | 0.0672 | 45 | 1 (ref) | 0.0211 | 175 | 1 (ref) | 0.3315 |
|  | 4795 | 334 | 1.15 (0.98; 1.35) |  | 73 | 1.66 (1.14; 2.42) |  | 193 | 1 (0.81; 1.23) |  |
|  | 4759 | 382 | 1.24 (1.05; 1.46) |  | 78 | 1.62 (1.09; 2.42) |  | 221 | 1.11 (0.9; 1.38) |  |
| RDW (%) ^d^ | 4775 | 258 | 1 (ref) | 0.0315 | 59 | 1 (ref) | 0.6867 | 145 | 1 (ref) | 0.0591 |
|  | 4937 | 329 | 1.05 (0.89; 1.24) |  | 62 | 0.96 (0.67; 1.38) |  | 201 | 1.14 (0.91; 1.42) |  |
|  | 4650 | 405 | 1.23 (1.04; 1.45) |  | 75 | 1.1 (0.76; 1.59) |  | 243 | 1.24 (0.99; 1.55) |  |
| **WBC** |  |  |  |  |  |  |  |  |  |  |
| WBC (10^9^cells/L) ^e^ | 4775 | 237 | 1 (ref) | <.0001 | 36 | 1 (ref) | 0.1016 | 151 | 1 (ref) | 0.1348 |
|  | 4734 | 297 | 1.04 (0.88; 1.24) |  | 73 | 1.49 (1.01; 2.21) |  | 167 | 0.89 (0.71; 1.11) |  |
|  | 4853 | 458 | 1.28 (1.08; 1.52) |  | 87 | 1.47 (0.98; 2.21) |  | 271 | 1.13 (0.9; 1.41) |  |
| Lymphocytes (109cells/L) | 4810 | 269 | 1 (ref) | 0.0208 | 58 | 1 (ref) | 0.647 | 151 | 1 (ref) | 0.2169 |
|  | 5037 | 321 | 1.02 (0.86; 1.2) |  | 62 | 0.97 (0.67; 1.4) |  | 197 | 1.1 (0.89; 1.37) |  |
|  | 4515 | 402 | 1.15 (0.97; 1.35) |  | 76 | 1.22 (0.85; 1.75) |  | 241 | 1.15 (0.93; 1.44) |  |
| Monocyte (109cells/L) | 4586 | 274 | 1 (ref) | 0.0001 | 54 | 1 (ref) | 0.0394 | 159 | 1 (ref) | 0.0552 |
|  | 5640 | 341 | 1.02 (0.86; 1.19) |  | 62 | 1.01 (0.69; 1.48) |  | 213 | 1.01 (0.82; 1.25) |  |
|  | 4136 | 377 | 1.23 (1.05; 1.44) |  | 80 | 1.26 (0.89; 1.79) |  | 217 | 1.17 (0.94; 1.45) |  |
| Neutrophils (109cells/L) | 4781 | 243 | 1 (ref) | 0.0006 | 44 | 1 (ref) | 0.3129 | 157 | 1 (ref) | 0.3552 |
|  | 4793 | 324 | 1.12 (0.94; 1.32) |  | 67 | 1.35 (0.91; 1.98) |  | 186 | 0.98 (0.79; 1.22) |  |
|  | 4788 | 425 | 1.22 (1.03; 1.44) |  | 85 | 1.39 (0.93; 2.06) |  | 246 | 1.08 (0.86; 1.34) |  |
| **Platelet** |  |  |  |  |  |  |  |  |  |  |
| Platelet (10^9^cells/L) | 4779 | 327 | 1 (ref) | 0.3811 | 60 | 1 (ref) | 0.2699 | 191 | 1 (ref) | 0.7178 |
|  | 4781 | 309 | 0.93 (0.8; 1.09) |  | 61 | 0.95 (0.67; 1.36) |  | 185 | 0.97 (0.79; 1.19) |  |
|  | 4802 | 356 | 1.04 (0.89; 1.21) |  | 75 | 1.21 (0.87; 1.71) |  | 213 | 1.03 (0.84; 1.26) |  |
| Plateletcrit (L/L) | 4798 | 301 | 1 (ref) | 0.0099 | 61 | 1 (ref) | 0.2225 | 179 | 1 (ref) | 0.2733 |
|  | 4774 | 307 | 1 (0.85; 1.17) |  | 55 | 0.96 (0.67; 1.38) |  | 187 | 1.02 (0.83; 1.26) |  |
|  | 4790 | 384 | 1.17 (1.01; 1.36) |  | 80 | 1.21 (0.86; 1.69) |  | 223 | 1.11 (0.9; 1.36) |  |
| MPV (fL) | 4836 | 331 | 1 (ref) | 0.6803 | 68 | 1 (ref) | 0.1696 | 198 | 1 (ref) | 0.5319 |
|  | 4724 | 317 | 0.95 (0.82; 1.11) |  | 69 | 0.93 (0.66; 1.31) |  | 194 | 0.99 (0.81; 1.22) |  |
|  | 4802 | 344 | 0.96 (0.82; 1.12) |  | 59 | 0.8 (0.57; 1.12) |  | 197 | 0.94 (0.76; 1.15) |  |
| PDW (%) ^f^ | 4652 | 309 | 1 (ref) | 0.8661 | 53 | 1 (ref) | 0.8145 | 190 | 1 (ref) | 0.1435 |
|  | 5010 | 345 | 0.98 (0.84; 1.14) |  | 74 | 0.99 (0.69; 1.43) |  | 207 | 0.94 (0.76; 1.15) |  |
|  | 4700 | 338 | 0.92 (0.79; 1.09) |  | 69 | 1.01 (0.7; 1.47) |  | 192 | 0.84 (0.68; 1.04) |  |

*Model adjusted for age, smoking status and intensity (7 categories), body mass index (BMI, continuous), Waist-to-hip ratio (WHR, continuous), physical activity level (Cambridge index, 4 categories), educational level (low, medium, high), alcohol intake (non drinker, occasional drinker, frequent drinker), systolic blood pressure, HDL cholesterol, diabetes. Stratified by sex and cohort center. Age is the underlying time variable. Exit age is age at first outcome of interest or censoring.

^a^ p-value for trend calculated modelling the median of each parameter in each tertile as a continuous variable

^b^ Red blood cells: further adjusted for haemoglobin; ^c^ Mean corpuscular volume; ^d^ Red cell distribution width: further adjusted for haemoglobin, RBC, WBC and platelet count; ^e^ White blood cells; ^f^ Platelet distribution width: further adjusted for platelet count;

Abbreviations: CVD, cardiovascular disease; CHD, coronary heart disease

**Supplemental Figure 1.** Meta-analysis of existing studies and the present results of the association between RDW and CVD risk


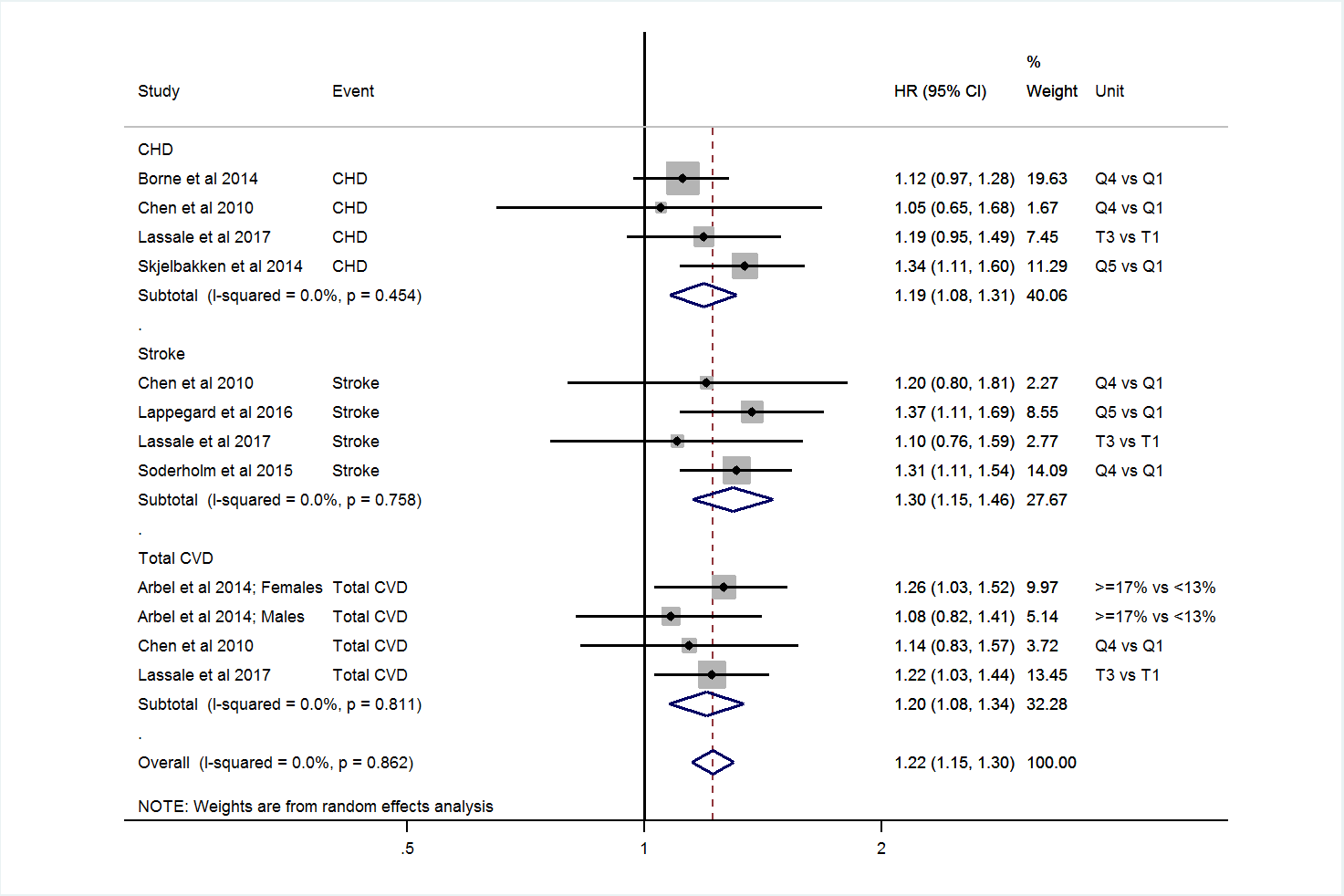

Supplement: Supplementary file 1 — Supplementary information [file 41598_2018_21661_MOESM1_ESM.doc]
